# Parthenolide promotes expansion of Nestin+ progenitor cells *via* Shh modulation and contributes to post-injury cerebellar replenishment

**DOI:** 10.3389/fphar.2022.1051103

**Published:** 2022-10-28

**Authors:** Dong Jinling, Feng Liyuan, Fu Wenying, Huang Yuting, Tang Xiangyu, Huang Xiuning, Tang Yu, Ming Qianliang, Guo Linming, Gao Ning, Li Peng

**Affiliations:** Department of Pharmacognosy and Traditional Chinese Medicine, College of Pharmacy and Laboratory Medicine, Army Medical University, Chongqing, China

**Keywords:** parthenolide, cerebellum, NEPs, Shh, irradiation

## Abstract

**Background:** Regeneration of injuries occurring in the central nervous system is extremely difficult. Studies have shown that the developing cerebellum can be repopulated by a group of Nestin-expressing progenitors (NEPs) after irradiation injury, suggesting that modulating the mobilization of NEPs is beneficial to promoting nerve regeneration. To date, however, effect of exogenous pharmaceutical agonist on NEPs mobilization remains unknown. Parthenolide (PTL), a sesquiterpene lactone isolated from shoots of feverfew. Although it has been shown to possess several pharmacological activities and is considered to have potential therapeutic effects on the regeneration of peripheral nerve injury, its efficacy in promoting central nervous system (CNS) regeneration is unclear. In this study, we aimed to elucidate the role and possible mechanism of PTL on regeneration in injured CNS after irradiation using a developing cerebellum model.

**Methods:** We investigated the radioprotective effects of PTL on the developing cerebellum by immunoblotting as well as immunofluorescence staining and ROS detection *in vivo* and *in vitro* experiments, and then determined the effects of PTL on NEPs in Nestin CFP and Nestin GFP fluorescent mice. Inducible lineage tracing analysis was used in Nestin-CreERT2×ROSA26-LSL YFP mice to label and track the fate of NEPs in the cerebellum after irradiation. Combined with cell biology and molecular biology techniques to determine changes in various cellular components in the cerebellum and possible mechanisms of PTL on NEPs mobilization in the injured developing cerebellum.

**Results:** We found that PTL could attenuate radiation-induced acute injury of granule neuron progenitors (GNPs) in irradiated cerebellar external granule layer (EGL) by alleviating apoptosis through regulation of the cells’ redox state. Moreover, PTL increased cerebellar Shh production and secretion by inhibiting the PI3K/AKT pathway, thus promoting expansion of NEPs, which is the compensatory replenishment of granule neurons after radiation damage.

**Conclusion:** Collectively, our results indicate that activation and expansion of NEPs are critical for regeneration of the injured cerebellum, and that PTL is a promising drug candidate to influence this process.

## Introduction

It is widely acknowledged that regeneration of injury to the central nervous system (CNS) is exceptionally rare, and that it causes irreversible and permanent damage. Over half of the human brain’s mature neurons are located in the cerebellum, although it represents only 10% of the total brain volume (Manto et al., 2020; De Zeeuw et al., 2021). Recent works have shown that the cerebellum plays a major role not only in maintaining motor coordination, but also in processing perception, cognition, and emotion (Van Overwalle et al., 2020). Consequently, it has become an excellent model for studying neurogenesis and circuit assembly, thereby attracting numerous research interest as a locus for a range of disorders and diseases (Beckinghausen and Sillitoe, 2019). Cerebellar growth occurs mainly after birth in mice and is due to rapid proliferation of Shh-driven external granule layer (EGL). Proliferation of cerebellar granule neural precursors (GNPs) in the EGL peaks at postnatal day 7 (P7) and ends at P15 as the GNPs exit the cell cycle and migrate to the internal granular layer (IGL), and differentiate into cerebellar granule neurons (Consalez et al., 2020). Studies have shown that although repeated of X-ray doses may cause cerebellar malformation at the proper time of pregnancy and during the early postnatal period, some granule neuron progenitors (GNPs) can survive, proliferate and reconstruct EGL under the action of a single X-ray dose (Altman et al., 1969). Recent experimental observations have revealed that a population of cerebellar Nestin-expressing precursors (NEPs) is responsible for regeneration of damaged developing cerebellum. NEPs can convert their differentiation capacity to produce mature granule neurons after irradiation injury, a process regulated by Shh signaling in the microenvironment. (Wojcinski et al., 2017). Correspondingly, in our previous study, a population of radiation-resistant precursor cells characterized by Nestin expression was also identified in the developing cerebellar EGL (Li et al., 2013), results that indicated that therapeutic strategies targeting NEPs have potential for regeneration of the developing cerebellum These results suggest that the development of therapeutic strategies for targeting NEPs is potentially valuable for regeneration of the developing cerebellum after injury. Despite its potential as a drug, reports have shown that the Shh protein has a neuroprotective effect, as evidenced by reduction of behavioral defects in a Parkinson’s disease rat model and induction of differentiation of dopaminergic neurons (Patel et al., 2017). However, Shh has a relatively short half-life in serum, and its curative effect is difficult to evaluate *in vivo*. In addition to playing a crucial role in cerebellar patterning, Shh also regulates normal cell development (Brady and Vaccarino, 2021). In particular, aberrant activation of Shh signaling can cause hyperproliferation and malignancy (Northcott et al., 2019). During neurodevelopment, in addition to the classical pathway, Shh signaling can support rapid cell growth by activating aerobic glycolysis and lipogenesis. This process is mediated by various genes including hexokinase 2 (HK2) and pyruvate kinase M2 (PKM2), and the upregulation of aerobic glycolysis is a distinctive feature of the developing cerebellum (Gershon et al., 2013; Tech et al., 2017). From a clinical perspective, there is need to prospect for agents that can cross the human blood-brain barrier, accurately and effectively control Shh signals in the microenvironment within a certain range, and also exert multiple protective effects for NEPs expansion through various pathways.

PTL, a sesquiterpene lactone mainly derived from the medicinal herb Feverfew (*Tanacetum parthenium*), has been widely utilized for treatment of high fever, headache, stomach pain, toothache, rheumatoid arthritis, irregular menstruation and other inflammatory diseases (Ghantous et al., 2013). Its rapid interaction with biological targets, anti-inflammatory, redox-modulating, and epigenetic activities, as well as selective cytotoxicity to cancer stem and progenitor cells, is regulated by the nucleophilic properties of methylene γ-lactone ring and epoxy group (Freund et al., 2020). Numerous studies have shown that PTL also has a protective effect on the nervous system, and can cross the blood-brain barrier to reduce brain inflammation (Zhang et al., 2022). The experimental results showed that PTL significantly improved neurological deficits, brain water content and infarct volume in a permanent middle cerebral artery occlusion (MCAO) model. (Dong et al., 2013). In an intracranial hemorrhage (ICH) rat model, PTL was also found to increase the number of surviving neurons to the improved neurological deficit and brain edema (Wang et al., 2020). Another study showed that PTL ameliorated oxygen-glucose deprivation ischemia/reperfusion-evoked neuronal injury and oxidative stress in PC12 cells (Zhang et al., 2017). Earlier researches focused on PTL’s protective effects on cytotoxicity by inhibiting NF-κB. Interestingly, Gobrecht and colleagues (Gobrecht et al., 2016) demonstrated that a single low-dose of PTL also significantly promoted regeneration of injured sciatic nerve axonal by interfering with detyrosination of alpha-tubulin in an NF-κB-independent manner. Furthermore, PKM2, a master regulator of aerobic glycolysis, is highly expressed in undifferentiated cerebellar GNPs, and aerobic glycolysis is involved in cerebellar development (Tech et al., 2017). Recent studies reveal that PTL and its derivatives as activators of PKM2 can promote tetramer formation of PKM2, prevent nucleus translocation of PKM2 dimer, and inhibited PKM2/STAT3 signaling pathway *in vitro* and *in vivo* (Ding et al., 2020; Liu et al., 2021). Collectively, these data indicate that PTL has multiple actions and a pharmacological potential for promoting nerve regeneration. Although PTL can increase the radiosensitivity of prostate cancer cells while preventing radiation damage to normal prostate ones (Morel et al., 2017), its neuroprotective effects in Irradiation-induced CNS injury, especially in regeneration of developing cerebellum, remains unclear.

Here, we report that PTL is a promising neuroprotectant that can protect GNPs from acute radiation injury by regulating oxidative stress levels of GNPs in EGL after radiation. Our results further show that PTL can also inhibit PI3K/AKT phosphorylation and increase secretion of Shh by cerebellar irradiated astrocytes to promote NEPs expansion. Collectively, our findings demonstrate that PTL is a potential alternative drug for regeneration of the developing cerebellum after injury.

## Materials and methods

### Animals and treatments

Five strains of mice were used in the experiment. Nestin-CFP mice were a kind gift from Yang Lab (The Fox Chase Cancer Center, Philadelphia, United States). Nestin-GFP mice were obtained from Cyagen (Suzhou, China). Nestin-CreERT2 and ROSA26-LSL YFP reporter mice were purchased from The Jackson Laboratory (Bar Harbor, ME). C57BL/6J mice were purchased from the Experimental Animal Center of the Army Medical University (AMU, Chongqing, China). The purchased mice were mated and bred, and newborn mice were obtained from their offspring for experiments. Juvenile mice were bred from these above. The adult mice used in the experiment were half males and half females. While some of the mice used were too young to distinguish between males and females. Mice receiving brain tissue died after perfusion, and non-perfused mice were killed by carbon dioxide inhalation after completion of the experiment. Animal was divided into several groups (Supplementary Table S1). All animal experiments were performed in compliance with institutional guidelines and had been approved by the Laboratory Animal Welfare and Ethics Committee of Army Medical University (approval number:AMUWEC20211295). All mice were raised in pathogen-free conditions, maintained at a temperature of 22°C–25°C, with a 12-h light/dark cycle, regular ventilation and free access to water and food, which approved all animal care and experimental procedures of the Experimental Animal Center in AMU. Animals that reached the end point were sacrificed by CO_2_ anesthesia.

Tamoxifen (TM; Sigma T5648) was dissolved in corn oil at 20 mg/ml. Nestin-CreERT2 × ROSA26-LSL YFP pups received intraperitoneal injections of 100 μg of TM on postnatal days P4 to induce Cre-mediated recombination. Parthenolide (PTL) was dissolved in dimethyl sulfoxide (DMSO) at 20 mg/ml. P4 mice were received one 40 mg/kg dose of PTL *via* intraperitoneally injection, 2 h later, mice were anesthetized by hypothermia and irradiated only in cerebellum (CB) for 4Gy using an X-RAD RS 2000 (Rad source Technologies) in the central Lab of AMU. To specifically target the CB, the rest of the body was covered with the lead plate. All chemicals were purchased from Sigma-Aldrich (St. Louis, MO).

### Tissue processing and immunofluorescence

During anesthesia, pentobarbital (50 mg/kg) was injected intraperitoneally, and ice-cold PBS and 4% paraformaldehyde were perfused transcardially to the animals. As described previously, brains were fixed overnight in 4% paraformaldehyde (PFA) at 4°C and then submerged in 30% sucrose until sinking (Li et al., 2013). Sagittal sections of 8 μm thicknesses were prepared using a cryostat (Thermo Fisher Scientific) at −20°C and stored at −80°C until use. For staining, Sections and cells were blocked with 10% goat serum (BeyotimeBiotech) in PBS containing 0.05% TritonX-100 for 1 h, incubated with various primary antibodies (Supplementary Table S2) overnight at 4°C, washed three times with PBST, and added with secondary antibodies (Supplementary Table S3), protected from light at room temperature Incubate for 2 h and then counterstain with DAPI (Solarbio). Images were captured using a Leica fluorescence microscope at 10X and 40X magnification (Leica, Germany). Images were analysed using Adobe Photoshop and ImageJ software.

### Granule neuron precursors isolation and cell culture

For GNPs isolation, we followed the previously described protocol (Li et al., 2013), cerebella were harvested from C57BL/6J mice at P4-P7 then digested in a solution containing 10 units/ml papain (Worthington), 25 U/ml DNase I (Solarbio) and 2 mg L-cysteine (Solarbio) at 37°C for 30 min to obtain a single-cell suspension. After filtered with 70 μm strainer, cells were centrifuged through a 35%–65% Percoll gradient (GE Healthcare). Cells from the 35–65% interface were suspended in Neurobasal Medium with B27 supplement. Then GNPs were suspended in NB-B27 and plated on Poly-L-omithine hydrobromide (PLO) and Laminin (all from Sigma)-coated coverslips. GNPs was treated with 5 μM PTL, 50% sonic hedgehog conditional medium (Shh-CM) and Shh-CM adding 5 μM PTL for 24 h. EDU (Proteintech) was added 20 μM and incubated GNPs for 1 h. Then stained according to the instructions.

### Primary astrocytes isolation and culture

Embryonic day 14.5 (E14.5) cerebella of C57BL/6J mice were used to prepare primary astrocytes. Isolation and culture of primary astrocytes were performed according to previous instructions (Liu et al., 2017). Briefly, cerebella were digested using 0.125% trypsin at 37°C for 15 min to obtain a single cell suspension, then plated on a 75 cm^2^ flask coated with poly-D-lysine (Solarbio) at a density of 20,000 cells/cm^2^. Cells were cultured in Dulbecco’s modified Eagle’s medium (DMEM, BI) containing 1% N2-Supplement-A (Stem cell), 1% fetal bovine serum (FBS, BI), 2 mM L-glutamine (Gibco), 100 U/mL penicillin and 0.1 mg/ml streptomycin (BI). For the following experiments, pure passage 2 to 5 was used. Astrocyte cell line C8-D1A was purchased from China Center for Type Culture Collection (Wu Han, China). C8-D1A were cultured in Dulbecco’s modified Eagle’s medium (DMEM, BI) containing 10% fetal bovine serum (FBS, BI), 100 U/mL penicillin and 0.1 mg/ml streptomycin (BI). Media was changed twice weekly.

### Determination of reactive oxygen species production

The commercial kit (Nanjing Jiancheng Institute of Biological Engineering) was used to determine the content of hydrogen peroxide in cerebellum. H_2_O_2_ combines with molybdic acid to form a complex, which was determined at the 405 nm wavelength, and the content of H_2_O_2_ was calculated according to the instructions.

The intracellular ROS levels were detected by Reactive Oxygen Species Assay Kit (Beyotime Biotechnology, China) according to the manufacturer’s protocol. Briefly, the cells were seeded in 96-well plates. After the treatment, the cells were incubated with 10 μM DCFH-DA for 20 min at 37°C. Then cells were measured at 488 nm excitation and 525 nm emission by a fluorescence spectrophotometer (BMG LABTECH, GRE).

### RNA extractions and quantitative RT-PCR

In accordance with the manufacturer’s protocol, total RNAs were extracted from cultured cells using Trizol reagent (DingGuo). cDNA was prepared using 5x HiScript II RT SuperMix (Vazyme). Quantitative PCR was performed using 2x AceQ qPCR SYBR Green Master Mix (Vazyme). In this study, fold-changes in expression were calculated based on the ΔΔCt method. Results were normalized using the GAPDH gene. The primer pairs were listed in Supplementary Table S4.

### Western blot

Cells or tissues were lysed in cell lysis buffer (Beyotime Biotechnology) on ice or liquid nitrogen to obtain protein lysate and the concentration measured using an enhanced BCA protein assay kit (Beyotime Biotechnology). Proteins were separated by sodium dodecyl sulfate polyacrylamide gel electrophoresis (SDS-PAGE) gels and transferred to polyvinylidene difluoride (PVDF) membranes (Merck Millipore), after blocking with 5% non-fat milk in Tris-buffered saline membranes were incubated with primary antibodies (Supplementary Table S5) overnight at 4°C, then incubated with secondary antibodies (Supplementary Table S6) at room temperature. To detect immunoreactivity, blots were incubated with Immobilon western chemilum hrp substrate (Millipore, WBKLS0100) and imaged on Fluorescence Chemiluminescence Imaging System (CLiNX). Relative intensity of blots was quantified using ImageJ software.

### Quantifications and statistical analyses

The area near the midline of the cerebellum (μm^2^) was measured using ImageJ software. For all IF staining, cell counts were obtained using ImageJ and Photoshop CS6. Certainly, three sections per animal, and at least 3 animals per group, were analyzed. Results were expressed as mean ± SEM. The statistical analyses were conducted using Prism version 6 (GraphPad Software) and SPSS version 20 (IBM Corp.). Two-tailed Student’s T tests were used to analyze the data in relation to vehicles or scrambled controls. When more than two groups were compared, Kruskal–Wallis nonparametric one-way ANOVA was used to detect group effects, followed by Dunn’s multiple comparison post hoc test. Significance was determined at *p* < 0.05. ns, not significant; *p < 0.05; **p < 0.01; ***p < 0.001.

## Results

### NEPs exhibit differential spatiotemporal distribution in neonatal mouse cerebellum

Wojcinski et al. (2017) used *in vivo* lineage-tracing Cre/loxP technologies to reveal that NEPs switched their fate from producing astroglia and interneurons during mid-embryogenesis to generate granule neurons in mice. Their concern, however, was how the existing NEPs could regenerate GNPs after irradiation damage. In the present study, we focused on the role of the newly produced NEPs in this process. To investigate distribution patterns of NEPs in the developing postnatal cerebellum, we studied Nestin-CreERT2 mice crossed with ROSA26-LSL YFP mice to obtain Nestin-CreERT2; ROSA26-LSL YFP (NR) mice, which expressed GFP preceded by a loxP-flanked stop sequence. We intraperitoneally injected tamoxifen into P0, P2, and P3, respectively, then analyzed distribution of NEPs cells in the cerebellum *via* GFP immunofluorescence staining. Results revealed that the number and distribution of fluorescently labeled cells (NEPs) in the cerebellum differed dramatically when Tamoxifen was given at different developmental stages. Most cells were labeled at P0, and positive cells were found in the cerebellar EGL, Purkinje cell layer (PCL), and IGL, of which PCL represented the site with the highest abundance (Supplementary Figure S1A). Delayed TAM administration resulted in a sharp decrease in number of fluorescently labeled NEPs, and the labeled cells in P2 and P3 pups were scattered in the EGL and PCL (Supplementary Figure S1B,C). These results suggest that NEPs gradually lose the ability to express Nestin with development and differentiation of the cerebellum, thus may be transformed into other cell types. Next, we investigated whether radiation affects the distribution of labeled cells induced by TAM by comparing expression patterns of GFP positive cells labeled by TAM before and after irradiation. Results showed that the number of TAM-labeled NEPs at P3 was significantly lower after irradiation than before, although both of them were irradiated in the cerebellum at P1, which indicated that there were not many new NEPs produced after irradiation (Supplementary Figure S1D,E). Furthermore, we explored new generation of NEPs in the developing cerebellum after irradiation at different stages. Interestingly, we found that a significantly higher number of new NEPs emerged in the P4-irradiated cerebellum compared to other periods (Supplementary Figure S1F). In order to have enough newly generated NEPs for the study at a later stage, we employed the irradiation strategy in P4 pups in the follow-up experiment.

### PTL protected the cerebellum from acute radiation injury by modulating cerebellar ROS levels

To exclude possible TAM effects on neuroprotection, we first confirmed the protective effect of PTL using wild-type mice. To this end, we employed PTL acute toxicity studies using a series of doses based on the *in vitro* results and previous literature (Freund et al., 2020; Wang et al., 2020) Firstly, weassessed thee effects of various PTL concentrations on proliferation of GNPs *in vivo*. Results from Ki67 immunofluorescence staining revealed that PTL had no significant effect on the proliferation of GNPs in the cerebellar EGL relative to the control sample even at the highest concentrations (i.e., nearly 40 mg/kg) (Supplementary Figure S2A,B). Next, we compared the effect of PTL administration schedule. To this end, we determined PTL’s protective effect before or after irradiation on the EGL of irradiated cerebellum, which has implications for application of PTL as a prophylactic or protective agent. Considering the single dose and the blood concentration across the blood-brain barrier (BBB), we used 40 mg/kg as the fixed dose for *in vivo* assay in our experiment. Analysis of IF results, 24 and 48 h after IR, showed that mice cerebellum pre-treated with PTL had more Ki67 + cells in the EGL than the vehicle after IR (*p* < 0.01, Supplementary Figure S2C–F). To confirm if high PTL concentrations were safe for the GNPs, we again subjected cerebellar sections to γ-H2AX staining after low (10 mg/kg) and high (40 mg/kg) dose administration and assessed for possible cellular DNA damage. Results showed that PTL did not increase γ-H2AX positive cells 4 h after irradiation, with high PTL concentrations associated with a decrease in the number of positive cells to some extent (Supplementary Figure S2G,H). Therefore, we selected 40 mg/kg PTL for 2 h before IR as the dosage in all subsequent experiments. Subsequently, we performed MTS assays to assess PTL’s cytotoxicity on primary GNPs, and found an IC50 value of 4.36 ± 0.68 μM (data not show). Consequently, we chose 1 μM PTL, which was lower than the IC50, for subsequent *in vitro* GNP experiments.

**FIGURE 1:**
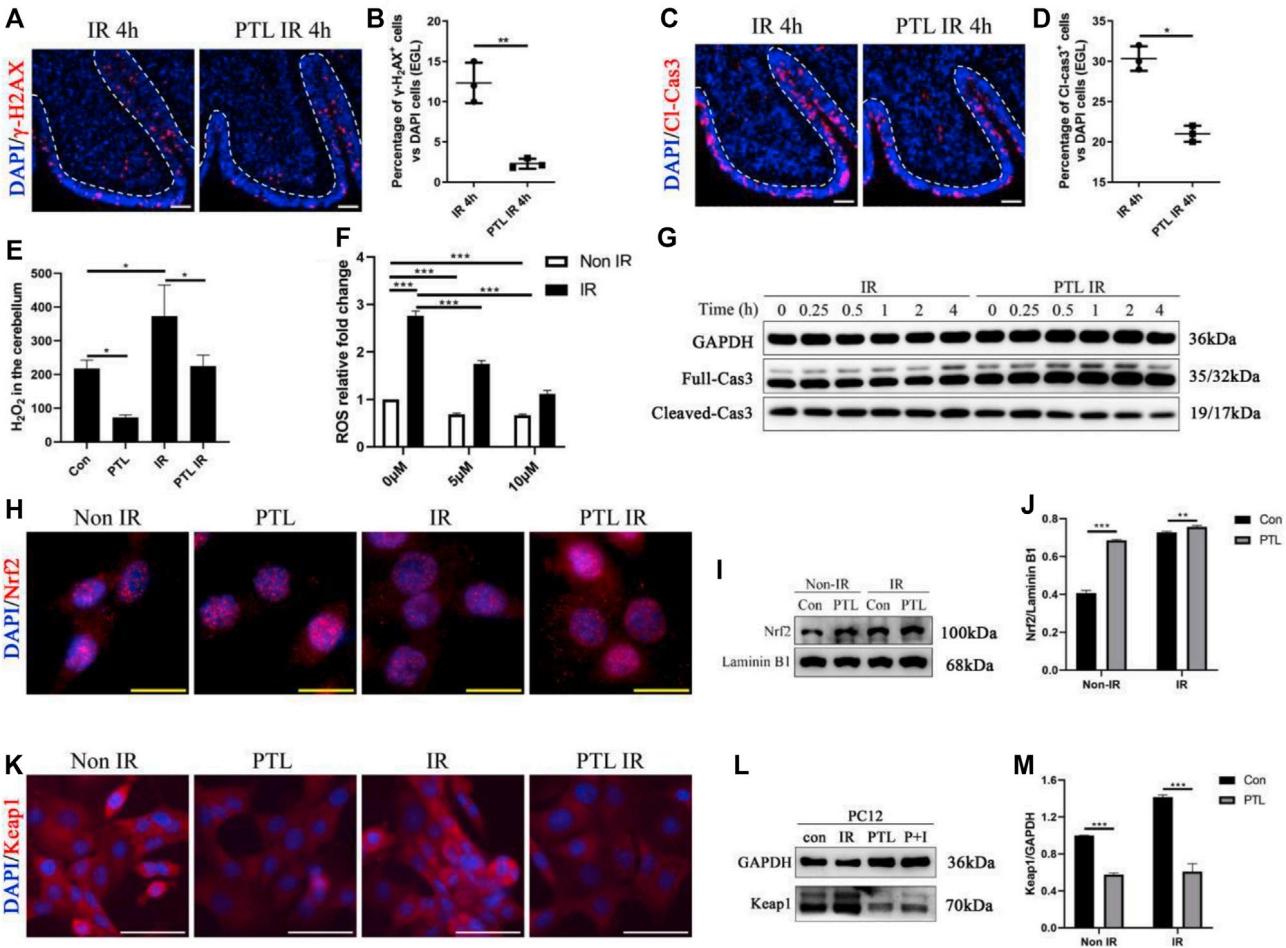
Pretreatment with PTL reduces cerebellar EGL loss after P4 irradiation by modulating cerebellar ROS levels. Sagittal sections, and stained for a marker of DNA damage (γ-H2AX; red), a marker of cell apoptosis (cleaved-caspase3, Cl-cas3; red), a marker of cell proliferation (Ki67; red) or nuclei (DAPI; blue). **(A)**. The γ-H2AX immunofluorescence detection of DNA damage (×100 magnification) on midsagittal sections (∼8 µm) of the cerebellum were taken from irradiated (IR) mice and PTL IR (PTL pretreated before irradiation) mice at corresponding time at P4 with red showing γ-H2AX foci and blue showing DAPI stained nuclei. The external granule cell layer (EGL) painted by white dotted line. **(B)**. Graph represents the quantifications of **(A)**. Each scatter points represented individual measurement of the percentage of γ-H2AX-positive cells in EGL of IR, PTL IR and IR PTL mice by 4 h after IR at P4 (n = 3). **(C)**. The Cl-cas3 immunofluorescence detection of apoptosis (×100 magnification) on midsagittal sections of IR and PTL IR mice by 4 h after IR at P4 with red showing Cl-cas3^+^ cells and blue showing DAPI stained nuclei. The external granule cell layer (EGL) painted by white dotted line. **(D)**. Graph represents the quantifications of **(A)**. Each scatter points represented individual measurement of the percentage of Cl-cas3-positive cells in EGL of IR, PTL IR and IR PTL mice by 4 h after IR at P4 (n = 3). **(E)**. Bar graphs represent the concentration of hydrogen peroxide (H_2_O_2_) in the cerebellum among Control (Con) mice, PTL treatment (PTL) mice, IR and PTL IR mice (n = 3). **(F)**. Bar graphs represent the DCFH-DA detection of ROS in PC-12 cells without radiation (non-IR, white) and with 4Gy X-ray radiation (IR, black) after pretreatment with 0 μM, 5 μM, and 10 μM PTL for 4 h (n = 3). **(G)**. PC-12 cells pretreated with or without 10 μM PTL for 1 h before 4 Gy X-ray exposure and were then irradiated. Representative Western blot images of GAPDH (top) and markers of apoptosis, total (middle) and cleaved Cas3 (down) at different time points after irradiation. **(H)**. Immunofluorescence analysis of the effect of 10 μM PTL preconditioning on changes of Nrf2 fluorescence (red, ×400 magnification) of PC-12 cells after 2 h of IR injury. Overlaying the images of Nrf2 and DAPI (blue fluorescence, used as a nucleus indicator) confirmed the nuclear location of Nrf2. **(I)**. Representative Western blot images of Nrf2 (top) and laminin B1 (for nuclear protein internal reference, down) in cells after 4Gy X-ray 2 h irradiation, non-PTL pretreated cells as control. **(J)**. Bar graphs represents the quantifications of **(I)**. Nrf2 was normalized to Laminin B1. Black bar, control; Gray bar, PTL pretreatment group (n = 3). **(K)**. Immunofluorescence analysis of the effect of 10 μM PTL preconditioning on expression of KEAP1 fluorescence (red, ×200 magnification) of PC-12 cells after 2 h of IR injury. DAPI (blue fluorescence) used as a nucleus indicator. **(L)**. Western blot analysis of GAPDH (top) and KEAP1 protein (down) in PC-12 cells pre-treated with 10 μM PTL or not after IR at 2 h **(M)**. Bar graphs represents the quantifications of (l). KEAP1 was normalized to GAPDH. Black bar, control (con, IR); Gray bar, PTL pretreatment (PTL, P + I) group (n = 3). All graphical data were presented as mean ± s.e.m., and significance was determined by two-tailed Student’s t test or Kruskal–Wallis nonparametric one-way ANOVAs. *p < 0.05; **p < 0.01; ***p < 0.001. White scale bar, 50 µm. Yellow scale bar, 20 µm.

We investigated the effect of PTL pretreatment on EGL GNPs damage and apoptosis after irradiation by analyzing levels of γ-H2AX and cleaved-caspase3 (Cl-cas3) expression. IF assay results showed that PTL IR mice had a lower proportion of γ-H2AX + cells (2.30 ± 0.62%) than their IR counterparts (12.33 ± 2.52%) (*p* < 0.01, Figures 1A,B), as well as lower proportions of Cl-cas3-positive cells (IR = 30.33 ± 1.53% vs. PTL IR = 21.00 ± 1.00%) (*p* < 0.01, Figures 1C,D). These results indicated that PTL could protect GNPs from acute radiation injury to a certain extent. Previous studies have shown that radiation-induced ROS production is an important factor determining radiation damage (Wozny et al., 2019). Next, we measured ROS levels in the cerebellum through H_2_O_2_ detection, and found that PTL pretreatment induced a 2.8- and 1.9-fold reduction in H_2_O_2_ production in PTL and PTL IR mice, respectively, relative to that in the control groups (Figure 1E). Since primary GNPs in the undifferentiated state cannot be cultured *ex vivo* for a long time, we used cell lines to study PTL’s effect on cell ROS production. Previous studies have demonstrated that PC12 cells derived from rat adrenal glands are an excellent model for studying differentiation of neuron precursors (He et al., 2020). In the present study, we performed DCFH-DA assay to detect ROS production in PC12 cells and found that irradiation mediated a 2.8-fold increase in cells. Notably, exposure to elevated PTL markedly reversed the observed increase in ROS production. ROS levels decreased to 64 and 21% in 5 μM and 10 μM PTL pretreatment groups, respectively. (Figure 1F). Next, we performed Western Blot assay to determine the rate of apoptosis in cells within 4 h of irradiation. Results showed that cleave-caspase 3 was significantly down-regulated in the PTL pretreatment group, although this change was not obvious in the single radiation group, possibly due to the short duration of exposure. These results suggest that PTL-mediated inhibition of apoptosis acts independently and are not initiated by irradiation (Figure 1G). Previous studies have shown that PTL can play an antioxidant role by regulating the Keap1-Nrf2 pathway (Xu et al., 2013). To assess the involvement of Keap1/Nrf2 in our model, we analyzed protein expression and intracellular distribution of Nrf2 and Keap1 after exposure to PTL. To this end, we used immunofluorescence (IF) and western blot assays to evaluate the capability of PTL to activate NRF2 in PC12. Staining results showed that Nrf2 was mainly expressed in the cytoplasm of control cells. However, irradiation induced its nuclear translocation to some extent, with PTL treatment further promoting this translocation (Figure 1H). Western blots revealed that PTL significantly upregulated Nrf2 expression, as evidenced by a 1.6-fold increase in the nucleus of PC12 cells treated with PTL for 2 h. Notably, PTL still generated a slight increase even under irradiated conditions (Figures 1I,J). On the other hand, Keap1 protein was mainly expressed in cytoplasm of PC12 cells, and KEAP1 staining was markedly diminished following treatment with PTL (Figure 1K). Western blots revealed a consistent pattern with IF results, relative to the untreated group. Notably, PTL downregulated KEAP1 expression by 48% in PTL treatment group and 61% in control group, respectively (Figure 1M). Collectively, these results indicated that PTL can protect the cerebellum from acute radiation injury by modulating cerebellar ROS levels through the KEAP1/NRF2 pathway.

### PTL attenuated cerebellar hypoplasia after irradiation injury

**FIGURE 2:**
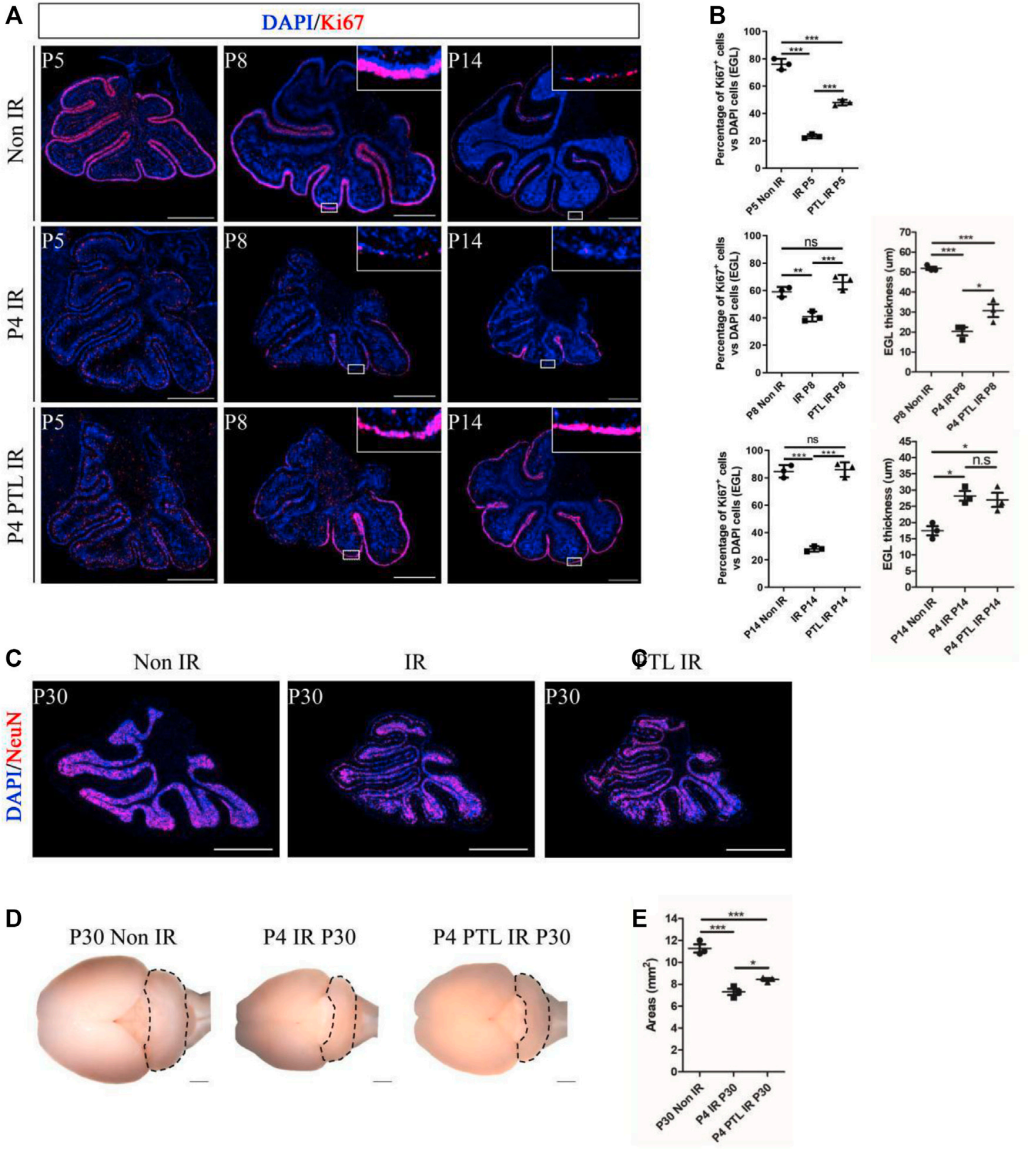
PTL promoted replenishment of the cerebellar EGL after irradiation damage. **(A)**. The Ki67 (red) immunofluorescence detection of proliferation (×50 magnification) on midsagittal sections of Non IR, IR and PTL IR mice at P5, P8, P14 with red showing Ki67^+^ cells and blue showing DAPI stained nuclei. High-power images were shown of the areas indicated by white rectangles. White scale bar, 500 μm. **(B)**. Graph showing the percentage of Ki67^+^ cells in EGL of Non IR, IR and PTL IR mice at P5, P8, P14. EGL Ki67^+^ cells decline significantly during IR, and PTL can relatively slow this decline at P5 (left/top, n = 3), P8 (left/middle, n = 3) and P14 (left/down, n = 3). Quantification of the EGL thickness showing thicker EGL in IR mice and lesser magnitude of thinning in PTL IR mice at P8 (right/top, n = 3). IR and PTL IR mice cerebella (right, n = 3). By P14, the thickness of EGL in IR mice and PTL IR mice was significantly higher than that in the Non IR mice (right/down, n = 3). **(C)**. IF detection of NeuN and DAPI on midsagittal sections (red, ×50 magnification) of Non IR, IR and PTL IR mice at P30. White scale bar, 500 μm. **(D)**. Bright field images of the cerebella of Non IR, IR and PTL IR mice cerebellum at P30. The cerebellum painted by black dotted line. **(E)**. Graph represents the quantifications of **(C)**. Each scatter points represented individual measurement of the areas of midline sections of the cerebella of Non IR, IR, and PTL IR mice at P30 (n = 3). All graphical data were presented as mean ± s. e.m., and significance was determined by two-tailed Student’s t test or Kruskal–Wallis nonparametric one-way ANOVAs. ns, nonsignificant; *p < 0.05; **p < 0.01; ***p < 0.001.

Cerebellum development is a highly coordinated process. After a short period of proliferation, GNPs in EGL migrate inward under the guidance of Bergman’s radial glial fibers and penetrate the internal granular layer (IGL), where they exit the cell cycle and complete cell differentiation. Restoration and maintenance of GNPs’ proliferative ability in EGL are crucial for regeneration of the damaged developing cerebellum (van der Heijden and Sillitoe, 2021). We first performed Ki67 staining to assess the effect of PTL pretreatment on GNPs proliferation at the different time points (P5, P8, P14) after IR on P4, and found that proliferating cells were stained red. Proliferating GNPs were mainly localized in the EGL on the cerebellar surface, and as the cerebellum developed, the cells exited the cell cycle and migrated inward, with the EGL layer becoming markedly thinner by P14, consistent with our earlier findings. Irradiation can cause destruction of EGL. After 24 h of irradiation, the EGL almost dissipated and only some scattered cells remained. After 4 days, the EGL recovered to some extent, but still did not return to normal levels. However, by P14, EGL had significantly thickened compared to the control group. In contrast, PTL pretreatment dramatically improved the damage to EGL at all stages of irradiation (Figure 2A). Next, we quantify cell proliferation by determining the percentage of Ki67-positive cells. Results showed that the rate of GNPs proliferation markedly decreased after 24 h, from 76.00 ± 4.00% to 23.33 ± 1.53% with IR, while PTL pretreatment restored it to 48.00 ± 2.00%. By P8, the proliferation rate declined from 59.00 ± 3.61% in the control to 41.00 ± 3.61% in the IR group and 66.00 ± 5.29% in PTL pretreatment. The remaining cells on the surface of normal cerebellar EGL showed a significant decrease at P14, although most of them kept proliferating. The percentage of Ki67 + cells in the IR group dropped to about 28.00 ± 2.00%, compared to about 84.67 ± 4.51% in normal mice. Pretreatment with PTL caused a considerable increase in the number of cells on the surface of EGL, while proliferation rates (86.00 ± 5.29%) returned to match those in untreated control cells. Furthermore, we compared the EGL thickness at P8 and P14, and found that PTL pretreatment could promote EGL replenishment after IR (Figure 2B). Notably, we found although irradiation caused a dramatic reduction in the thickness of cerebellar EGL at P8, nevertheless, the pretreatment of PTL (30.70 ± 5.61 μm) still performed slightly better than the pretreatment of IR (20.33 ± 3.59 μm). IR mice exhibited a thicker EGL at P14 (28.21 ± 2.59 μm) than their Non-IR (17.42 ± 2.49 μm) and PTL IR (26.96 ± 3.80 μm) counterparts. Collectively, these results indicated that IR disrupted proliferation and differentiation of GNPs, while PTL significantly ameliorated the proliferation inhibition of IR-induced GNPs. Previous studies had demonstrated that although the developing cerebellum can accomplish self-repair after IR, the volume of the repaired cerebellum after maturation is significantly smaller than that of a normal cerebellum (Wojcinski et al., 2017). In the present study, we measured the area of the sagittal section of the mature cerebellum (P30) and found that IR decreased the size of the cerebellum (7.30 ± 0.50 mm^2^) relative to the Non-IR (11.27 ± 0.64 mm^2^) group. However, PTL pretreatment rescued the cerebellar size (PTL IR = 8.43 ± 0.21 mm^2^) (Figures 2C–E). Taken together, these findings indicate that PTL sustained the proliferation of GNPs in EGL after IR, thus enhancing replenishment of the injured cerebellum.

### PTL promoted NEP reprogramming in both EGL and PCL of the cerebellum after IR

**FIGURE 3:**
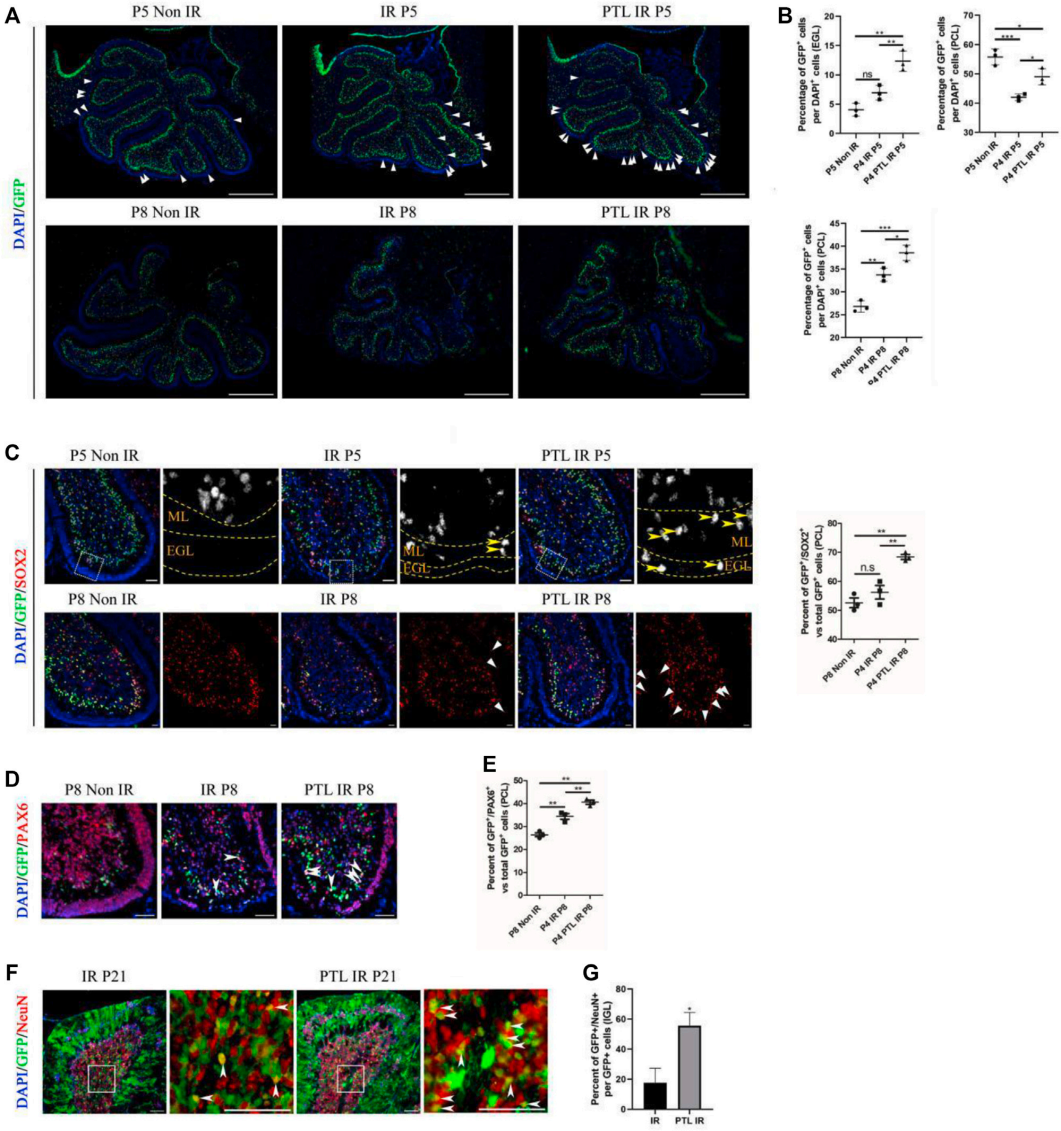
PTL augmented NEPs in both EGL and PCL of the cerebellum after IR Midsagittal sections (8 µm) of cerebellum were taken from Non IR, IR and PTL IR mice at indicated ages with the indicated transgene mice, and stained for GFP (green), Ki67 (red), a neuron stem cell marker (SOX2; red), a granule neuron precursors marker (PAX6; red) and DAPI (blue). a-e were Nestin-CFP mice. f-g were Nestin creER^T2^×Rosa26 LSL YFP (NR) mice injected with Tm at P5. **(A)**. The GFP immunofluorescence detection of NEPs (×50 magnification) on midsagittal sections of Non IR, IR and PTL IR mice at P5 (top) and P8 (down). The triangle indicated GFP^+^ cells in EGL. White scale bar, 500 μm. **(B)**. Graph represents the quantifications of **(A)**. Each scatter points represented individual measurement of the percentage of GFP + cells in EGL (top/left) and PCL (top/right) of Non IR, IR and PTL IR mice at P5 (n = 3). Scatter plot of the percentage of GFP + cells in PCL of Non IR, IR and PTL IR mice at P8 (down/left = 3). **(C)**. IF detection of GFP, SOX2 and DAPI on midsagittal sections (×100 magnification) of Non IR, IR and PTL IR mice at P5 and P8. High-power images were shown of the areas indicated by white dotted rectangles. The GFP^+^/SOX2^+^ cells are represented in grayscale for better visualization. Yellow arrowheads indicated GFP^+^/SOX2^+^ cells in EGL and ML (left/top). The left/down panel showing the staining at P8. White triangles indicated SOX2^+^ cells in EGL. White scale bar, 50 µm. Scatter plot of represents the percentage of GFP^+^/SOX2^+^ cells vs. all GFP^+^ cells in PCL of Non IR, IR and PTL IR mice at P8 (right, n = 3). **(D)**. IF detection of GFP, PAX6 and DAPI on midsagittal sections (×200 magnification) of Non IR, IR and PTL IR mice at P8. White arrowheads indicated GFP^+^/PAX6^+^ cells in PCL. White scale bar, 50 μm. **(E)**. Graph represents the quantifications of **(D)**. Each scatter points represented individual measurement of GFP^+^/PAX6^+^ cells vs. all GFP^+^ cells in PCL of Non IR, IR and PTL IR mice at P8 (n = 3). **(F)**. Nestin creER^T2^ postnatal lineage tracing of IR and PTL IR mice. IF detection of GFP, NeuN and DAPI on midsagittal sections (×200 magnification) of mice at P21. High-power images were shown of the areas (IGL) indicated by white dotted rectangles. White arrowheads indicate GFP^+^/NeuN^+^ cells in IGL. White scale bar, 50 μm. **(G)**. Graph represents the quantifications of **(F)**. Each scatter points represented individual measurement of percentage of GFP^+^/NeuN^+^ cells vs. all GFP^+^ cells in IGL of IR and PTL IR mice at P21 (n = 3). All graphical data were presented as mean ± s. e.m., and significance was determined by two-tailed Student’s t test or Kruskal–Wallis nonparametric one-way ANOVAs. ns, nonsignificant; *p < 0.05; **p < 0.01; ***p < 0.001.

Considering the plasticity of NEPs and the cell compensation mechanism after IR, which enhances recovery of the damaged cerebellum (Li et al., 2013; Wojcinski et al., 2017), we utilized Nestin-CFP transgenic mice to test the effect of PTL on NEPs. This choice was determined empirically in our previous studies (Li et al., 2013). Here, we selected P5 and P8 (24 and 96 h after IR) as phases for determining the amount of NEPs in EGL and PCL, respectively (Figures 3A,B). Firstly, we analyzed differences in CFP-positive cells in cerebellar EGL after about 24 h of irradiation (P5), and found that there were only a few CFP-positive cells in the residual EGL after irradiation, and although there was an increase in the proportion, it was not statistically significant (Non-IR = 4.05 ± 1.10% vs IR = 6.96 ± 1.24%). We attributed this phenomenon to a severe damage of EGL, which induced death of many cells whose nuclei subsequently stained positive for DAPI, thus lowering the ratio. PTL pretreatment promoted the increase in CFP positive cells in EGL after IR (PTL IR = 12.35 ± 1.75%). Next, we analyzed changes in CFP-positive cells in PCL, and found that IR decreased GFP expression in PCL (42.03 ± 1.16%) relative to non irradiated controls (55.79 ± 2.72%). PTL slightly reduced this IR-induced decrease (49.05 ± 2.78%). Comparison between CFP-positive cells in EGL and PCL of P8 mice revealed that CFP positive cells were scattered in all three groups of mouse EGL, albeit at no statistically significant difference. To this end, we hypothesized that NEPs would no longer express Nestin after differentiating into other types of cells, and its CFP fluorescence could not be detected. However, IR increased the number of CFP-positive cells in PCL (IR = 33.73 ± 1.43%) relative to the non-IR group (26.81 ± 1.25%). PTL contributed to the rise of CFP-positive cells in irradiation-induced PCL (PTL-IR = 38.54 ± 1.68%). Overall, these results indicated that IR can activate the mobilization of NEPs, a process that is accelerated by PTL pretreatment.

Studies had shown that NEPs can only differentiate into GABAergic interneurons and astrocytes under normal conditions, but cannot transform glutamatergic neurons (like granule neurons). Moreover, irradiation has been shown to induce NEP reprogramming in the cerebellum thus conferring them with the ability to differentiate into GNPs and mature granule neurons (Wojcinski et al., 2017; Andreotti et al., 2018). To determine whether PTL can promote NEPs reprogramming and enhance their ability to differentiate into GNPs, we stained GFP/SOX2 and GFP/PAX6 in Nestin-CFP mice. SOX2, a transcription factor of neural stem cells that regulates vertebrate neurodevelopment and adult neurogenesis (Wakamatsu and Uchikawa, 2021), is one of the key genes used to reprogram differentiated cells into induced pluripotent stem cells. Previous studies have also shown that SOX2-positive cells are involved in repairing damaged cerebellar EGL (Takahashi and Yamanaka, 2016; Wojcinski et al., 2017). In the present study, immunofluorescence staining results revealed that SOX2-positive cells were mainly distributed in PCL and WM under normal conditions. However, the number of CFP-positive cells in PCL decreased after 24 h of irradiation, while there were no significant differences in the number of SOX2 and CFP double-positive cells between the IR and PTL-IR groups. Moreover, irradiation induced appearance of SOX2 and CFP double-positive cells in the molecular layer, which is consistent with the previous report. Pretreatment with PTL resulted in a significant increase in the number of SOX2+/CFP + cells in the ML and EGL. By P8, there were about 51.4% SOX2+/CFP + cells in the PCL of control mice (non-IR) and 54.6% in IR mice, although there was no statistically significant difference between them. However, PTL pretreatment significantly increased double-positive cells in the PCL of PTL pretreated mice, reaching 67.8% (Figure 3C). Next, we evaluated the ability of NEPs cells to differentiate into granule neuronal cells by detecting PAX6 expression, a marker of granule neuron precursors (Park et al., 2018) in NEPs. At P5, we found no significant difference in expression of CFP+/PAX6+ cells among mice in Non-IR, IR, and PTL IR groups (Supplementary Figure S3A). In P8 Nestin-CFP transgenic mice, IR mediated upregulation of CFP+/PAX6+ cells in PCL (IR = 34.40 ± 2.12%), relative to Non-IR (Non-IR = 26.41 ± 1.51%) group (Figures 3D,E). In addition, we verified that PTL could promote NEPs mobilization in two other animal models and obtained similar results in Nestin-GFP (Supplementary Figure S3B) and Nestin-CreERT2/ROSA26-LSL YFP (NR) mice. In addition, we verified the fate of newly produced NEPs by activating Cre activity in NR mice with tamoxifen (Tm) 24 h after irradiation of P4 mice, and found pretreatment with PTL increased GFP+/PAX6+ cells in PCL after IR (IR = 10.67 ± 3.06%, PTL = 72.00 ± 8%) (Supplementary Figure S3C,D). These results indicated that PTL accelerated reprogramming of NEPs in EGL and PCL after irradiation.

To further understand the fate of NEPs affected by PTL, we crossed Nestin-CreERT2 mice to those harboring R26R-YFP to lineage-trace NEPs in the postnatal cerebellum. Next, we performed double staining of GFP and NeuN on cerebellar sections, at P21 (maturation stage of the cerebellum) and found that the cerebellar surface had abundant fiber-like green fluorescence-positive cells with a morphology similar to that of radial glial cells. The cerebellum’s internal IGL was filled with NeuN-positive cells, and the red fluorescence was mainly localized in the nucleus, which also had scattered green fluorescence-positive cells, both of which were able to co-localize. Results of the IF assay showed that PTL pretreatment promoted the expression of GFP+/NeuN + cells in IGL (IR = 18.23 ± 8.54% vs. PTL = 53.09 ± 6.74%) (Figures 3F,G). Taken together, our results demonstrated that PTL can not only induce mobilization but also contribute to differentiation of NEPs into neuronal lineage cells for the recovery of the injured cerebellum.

### Effects of PTL on interneurons and glial cells in irradiated cerebellar microenvironment

**FIGURE 4:**
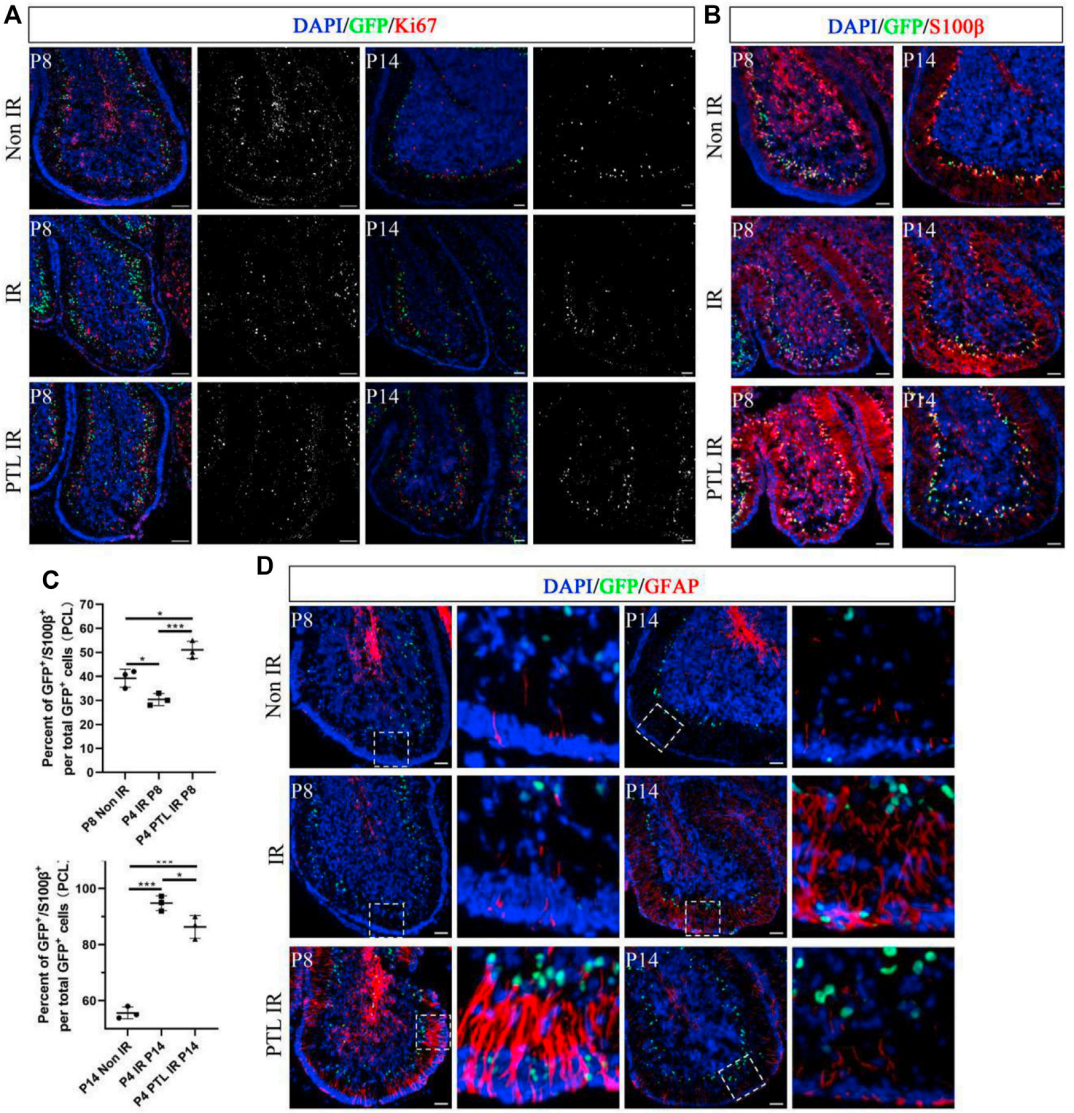
PTL facilitates the increase of cerebellar interneurons and glial cells after irradiation Midsagittal sections (8 µm) of cerebellum were taken from Non IR, IR and PTL IR mice at P8 and P14 with Nestin-CFP transgene mice. Stained for GFP (green), interneuron marker (PAX2; red), astrocytes marker (S100β; red) and glial filaments marker (GFAP; red) or DAPI (blue). **(A)**. IF detection of GFP, PAX2 and DAPI on sagittal sections (×100 magnification) of Non IR, IR and PTL IR mice at P8 (left two columns) and P14 (right two columns). Grayscale images indicated PAX2 positive cell. White scale bar, 50 μm. **(B)**. IF detection of GFP, S100β and DAPI on sagittal sections (×100 magnification) of Non IR, IR and PTL IR mice at P8 and P14. White scale bar, 50 μm. **(C)**. Graph represents the quantifications of **(B)**. Scatter plot of represents the percentage of GFP^+^/S100β^+^ cells vs. all GFP^+^ cells in PCL of Non IR, IR and PTL IR mice at P8 (left column, n = 3) or P14 (right column, n = 3). **(D)**. IF detection of GFP, GFAP and DAPI on sagittal sections (×100 magnification) of Non IR, IR and PTL IR mice at P8 (left two columns) and P14 (right two columns). Grayscale images indicated PAX2 positive cell. High-power images were shown of the areas indicated by white dotted rectangles. White scale bar, 50 µm. All graphical data were presented as mean ± s.e.m., and significance was determined by two-tailed Student’s t test or Kruskal–Wallis nonparametric one-way ANOVAs. ns, nonsignificant; *p < 0.05; ***p < 0.001.

The appropriate ratio of different types of cells in the cerebellum correlates with its function. Normally, the initial NEPs population in EGL mainly produces interneurons and astrocytes (Wojcinski et al., 2017). Previous results also confirmed that PTL pretreatment enhanced the production of radial glial cell-like cells from NEPs. Therefore, we investigated whether PTL had an effect on interneurons and glial cells of irradiated cerebellum. A comparison of the immunofluorescence expression profile of the interneuron marker PAX2 in the cells of NEPs at P8 and P14 revealed that CFP+/PAX2+ cells were scattered in the PCL of all three groups of mice, but their proportions were not significantly different. However, for PAX2+ cells, irradiation could reduce PAX2+ cells at P8. Although normal PAX2+ cells levels could not be restored, PTL pretreatment attenuated the reduction. At P14, the number of PAX2+ cells were significantly more than that in the control group, and the increase in positive cells in the cerebellum of PTL-pretreated mice was more pronounced compared with irradiation alone mice (Figure 4A). Ptf1a is a key transcription factor in the genesis of Purkinje cells and cerebellar GABAergic interneurons (Jin and Xiang, 2019). Quantitative PCR (qPCR) results revealed that PTL promoted the expression of Ptf1a, suggesting its role in differentiation of interneurons (Supplementary Figure S4A). We further investigated the change of astrocytes after treatment with PTL. IF assay showed that IR decreased the number of GFP+/S100β+cells in PCL (Non-IR = 39.26 ± 3.74%, IR = 30.36 ± 2.51%), but these were then increased by PTL treatment (PTL-IR = 51.04 ± 3.59%). At P14, IR increased the number of GFP+/S100β+cells in PCL (Non-IR = 55.63 ± 2.13%, IR = 94.76 ± 2.64%), but these were then slightly decreased by PTL (PTL-IR = 86.32 ± 4.04%). At this moment, astrocytes in PTL-IR mice and Non-IR mice showed similar morphology (Figures 4B,C). We tested the expression of the glial fibrillary acidic protein (GFAP), an important astrocyte marker expressed in the glial filaments. At P8, after IR treatment, filaments in EGL and ML were short and small, whereas PTL pretreatment induced the expression of GFAP and produced long and orderly filaments. At P14, IR still made the filaments disorderly, and PTL pretreatment made them perpendicular to the surface of EGL (Figure 4D). These results showed that PTL maintained the integrity of the filaments and may promote migration of GNPs in EGL after IR. We evaluated the effect of PTL on Purkinje cells utilizing Calbindin staining, a specific marker for Purkinje cells. At P8, Purkinje cells (PCs) from non-IR mice aligned in a monolayer, IR misaligned the PCs, but PTL pretreatment made them to align in a monolayer with richly arborizing planar dendrites. P14 showed that Purkinje cells were disordered in IR mice, whereas Purkinje cells were well-ordered in non-IR mice and PTL-IR mice (Supplementary Figure S4). These results suggest that PTL potentially has the ability to promote the maturation of post-IR Purkinje cells and maintain the orderly arrangement of post-IR Purkinje cells.

### PTL promotes the secretion of Shh in the cerebellum after IR

**FIGURE 5:**
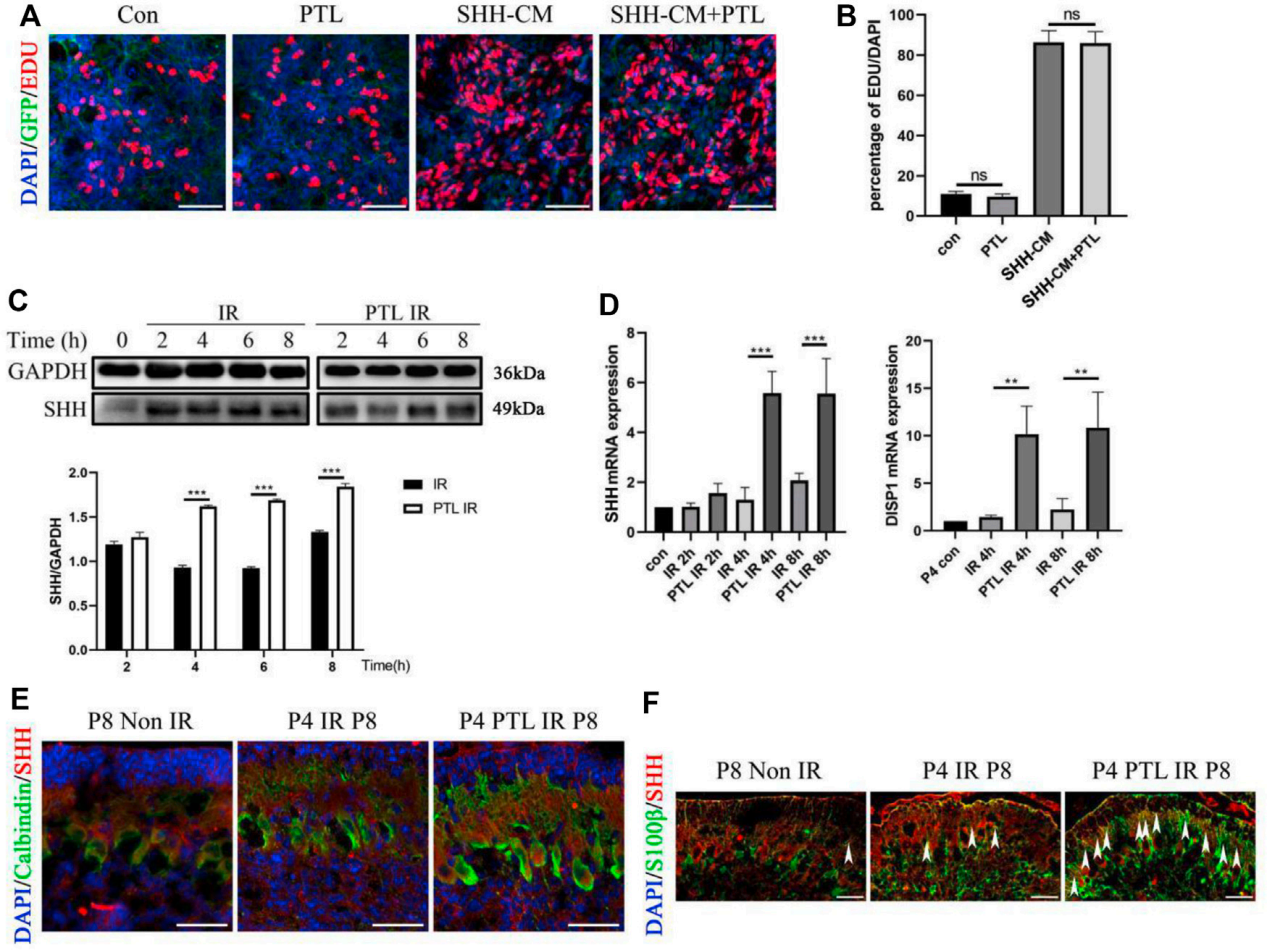
PTL promote Irradiation-Induced Production and Secretion of Shh in cerebellum **(A)**. Representative pictures (×100 magnification) of EdU and Tuj-1staining of GNPs. GNPs from wild type (WT) mice treated with 1 μM PTL, 50% Shh-CM, 50% Shh-CM adding 1 μM PTL *in vitro* for 24 h, then stained by β3-Tubulin (Tuj1; green) for neuron differentiation, EDU for proliferation (red) and DAPI (blue) for nuclei. White scale bar, 50 μm. **(B)**. Graph represents the quantifications of **(A)**. Scatter plot of represents the percentage of EDU^+^ cells vs. all DAPI cells of WT GNPs treated with 1 μM PTL, 50% Shh-CM, 50% Shh-CM adding 1 μM PTL (n = 3). **(C)**. Representative Western blot images of GAPDH and Shh from the wild type mice cerebella protein pre-treated with 40 mg/kg PTL or not at 2, 4, 6, 8 h after irradiation with 4Gy X-ray (top). Bar graphs represents the quantifications of Western blotting results. Shh was normalized to GAPDH. Black bar, IR; white bar, PTL IR group (down, n = 3). **(D)**. QRT-PCR quantification of Shh (left) and DISP1 (Dispatched-1, a membrane protein facilitates the release of Shh,right) from the cerebella of wild type mice pre-treated with 40 mg/kg PTL or not at 2, 4, 8 h after irradiation with 4Gy X-ray at P4 (n = 3). **(E)**. IF detection of Calbindin (Green), Shh (red) and DAPI (blue) on sagittal sections of Non IR, IR and PTL IR mice at P8 (×200 magnification). White scale bar, 20 μm. **(F)**. IF detection of S100β (Green), Shh (red) and DAPI (blue) on sagittal sections of Non IR, IR and PTL IR mice at P8 (×200 magnification). White arrowheads indicated S100β^+^/Shh^+^ cells present in PCL. White scale bar, 20 µm. All graphical data were presented as mean ± s. e.m., and significance was determined by two-tailed Student’s t test or Kruskal–Wallis nonparametric one-way ANOVAs. ns, nonsignificant; **p < 0.01; ***p < 0.001.

Considering the crucial role of Shh signaling in the development of normal cerebellum and the repair of damaged cerebellum, especially for the initial step in NEP reprogramming, we speculated that PTL might be a suppressor or activator of the Shh pathway. To test this hypothesis, we performed EdU proliferation incorporation assay to compare the proliferation of primary GNPs cultured under Shh-conditioned culture medium with or without PTL. If PTL had an effect on the SHH pathway, it would significantly affect the proliferation of Shh-induced GNPs cells *in vitro*. The proliferating cells, differentiated cells and nuclei were labeled with EdU (red), TUJ1 and DAPI (blue), respectively (Figure 5A). The results showed that about 11.3% of GNPs maintained their proliferation *in vitro* under normal conditions, and PTL had no significant effect on cell proliferation rate, which was about 10.5%. Stimulation of the Shh pathway increased the proportion of Edu-positive cells in GNPs to 82.6%, which was not significantly affected by PTL, with a rate of about 81.4% (Figure 5B). Our results showed that PTL had no apparent role on Shh signal transduction. Subsequently, we investigated the effects of time of PTL pretreatment on Shh protein expression in irradiated cerebellum. Interestingly, WB results showed that Shh was upregulated in the cerebellum after irradiation, and more significantly upregulated after PTL treatment in a time-dependent manner. There was no significant difference in Shh expression in the PTL pretreatment group compared with the irradiated group at 2 h. At 4 h, 6 h and 8 h, Shh expression level in the PTL pretreatment group was 1.6, 1.8 and 1.4 times higher than that in the irradiated group alone, respectively (Figure 5C). The same conclusion was reached using qPCR to observe Shh mRNA level. At 4 h and 8 h, the expression of Shh mRNA in PTL pretreatment group was 5.7 and 2.8 times higher than that in the irradiation group, respectively. DISP1 is required for secretion of lipid-modified Shh (Wang et al., 2015). PTL pretreatment increased DISP1 mRNA expression 9.6-fold and 5.8-fold in the 4 h and 8 h groups, respectively (Figure 5D). It was demonstrated that Shh is mainly made by Purkinje cells in the cerebellum (van der Heijden and Sillitoe, 2021). Therefore, we detected the expression of Shh in Purkinje cells by staining with Calbindin and Shh. IF assay showed that PTL IR mice had a stronger fluorescence of Shh in Purkinje cells at P8 (Figure 5E). Recently, many of researchers have found that astrocytes could also secrete Shh to support brain development (Liu et al., 2017; Petrov et al., 2017). Therefore, we also stained the astrocyte marker S100β and Shh in the frozen section. The results showed that PTL pretreatment increased the number of S100β+/Shh + cells after IR at P8 (Figure 5F). In general, PTL could promote Shh secretion in the cerebellum after IR.

### PTL facilitates secretion of Shh in astrocytes and protects cells from irradiation *via* the AKT pathway

**FIGURE 6:**
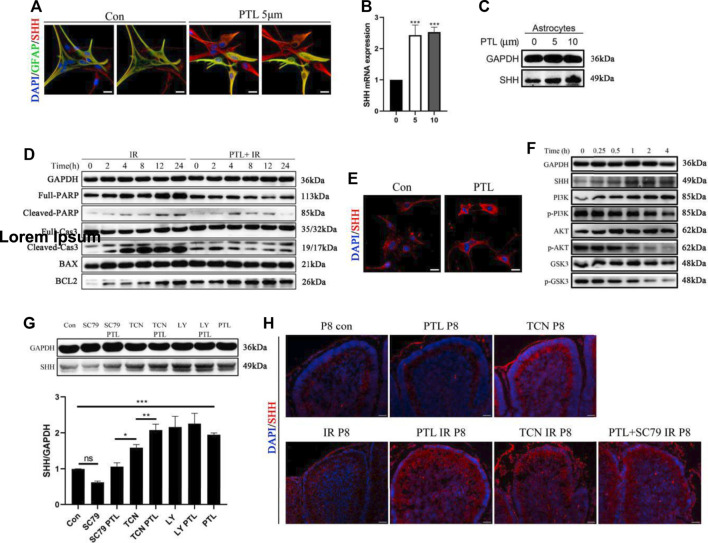
PTL increases the secretion of Shh in astrocytes through AKT pathway inhibition **(A)**. IF detection of GFAP (Green), Shh (red) and DAPI (blue) in primary astrocytes (×200 magnification) treated with or without 5 μM PTL for 24 h. Left two images are control, and the image on the right is without DAPI. Right two images are PTL treatment group, and the image on the right is without DAPI. White scale bar, 50 μm. **(B)**. QRT-PCR of Shh from primary astrocytes treated with or without 5 μM or 10 μM PTL for 24 h. Shh was normalized to β-actin. Black bar, control (0); black bar, 5 μM PTL pretreatment (5) Gray bar, 10 μM PTL pretreatment (10) (n = 3). **(C)**. Representative Western blot images of GAPDH and Shh from primary astrocytes treated with or without 5μM and 10 μM PTL for 24 h **(D)**. Astrocyte cell line (C8-D1A) pretreated with or without 10 μM PTL for 1 h before 4 Gy X-ray exposure and were then irradiated. Representative Western blot images of GAPDH and markers of apoptosis, PARP/p-PRAP, total Cas3/cleaved Cas3, BAX, BCL2 at different time points after irradiation. **(E)**. IF detection of Shh and DAPI in C8-D1A (×200 magnification) treated with or without 5 μM PTL for 24 h. White scale bar, 20 μm. **(F)**. Representative Western blot images of GAPDH, Shh, PI3K, p-PI3K, AKT, p-AKT, GSK3, p-GSK3 in astrocyte cell line (C8-D1A), treated with 10 μM PTL for 0, 0.25, 1, 2, 4 h **(G)**. Western blot of Shh in C8-D1A treated with 10 μM SC-79 (AKT activator), 10 μM SC-79 adding 5 μM PTL, 0.5 μM Triciribine (TCN, AKT inhibitor), 0.5 μM TCN adding 5 μM PTL, 1 μM LY294002 (LY, PI3K inhibitor), 1 μM LY adding 5 μM PTL for 24 h (top). Bar graphs represents the quantifications of Western blotting results. Shh was normal ized to GAPDH. (Down, n = 3). **(H)**. IF detection of Shh (red) and DAPI (blue) on sagittal sections of Con, PTL, TCN, IR, PTL IR, TCN IR and PTL adding SC-79 IR mice at P8 (×200 magnification). White scale bar, 20 µm. All graphical data were presented as mean ± s.e.m., and significance was determined by two-tailed Student’s t test or Kruskal–Wallis nonparametric one-way ANOVAs. ns, nonsignificant; *p < 0.05; **p < 0.01; ***p < 0.001.

In cerebellar radiation damage, the most affected cells by irradiation are granule neurons and astrocytes. Astrocytes were considered another potential primary cellular source of Shh (Alvarez et al., 2011). Therefore, we explored the effect of PTL on astrocytes. To test the effect of PTL on astrocytes *in vitro*, we used primary astrocytes from wild-type mice embryonic day 14.5 (E14.5). After Shh staining in comparison with the control group without PTL treatment, stronger red fluorescence (Shh) was found in primary astrocytes treated with PTL for 24 h. Astrocytes were labelled with immunofluorescent green for GFAP. Although GFAP expression in primary astrocytes was heterogeneous, including weak, moderate and strong positive staining, we could still observe co-localization of GFAP with Shh (yellow) (Figure 6A). qPCR results showed that 5 μM and 10 µM PTL treatments increased Shh mRNA expression in primary astrocytes approximately 2.3 and 2.5-fold, respectively, which was consistent with WB result (Figures 6B,C). To study the mechanism by which PTL protects astrocytes, we utilized the astrocyte cell line C8-D1A for this experiment. First, to determine whether PTL could protect astrocytes after irradiation *in vitro*, we irradiated C8-D1A cells at 4Gy X-ray pre-treated with 10 μm PTL for 2 h. After IR-activated cell apoptosis, the DNA repair enzyme poly (ADP-ribose) polymerase (PARP) was cleaved to p-PARP by Caspase 3 (Cas3), which was simultaneously transformed to Cl-cas3. BCL2 plays an antiapoptotic role in this process, whereas BAX not only antagonizes the inhibitory effect of BCL2, but also promotes apoptosis. WB assay showed that pretreatment with PTL significantly inhibited the expression of PARP, p-PARP, Cl-cas3, and BAX, but promoted the expression of BCL2 (Figure 6D). These results suggest a protective role of PTL for irradiated astrocytes *in vitro*. Immunofluorescence assay showed that PTL also increased the expression of Shh in C8-D1A cells (Figure 6E). Acute neuronal molecules glycogen synthase kinase-3β (GSK-3β) and phosphatidylinositol-3 kinase/AKT (PI3K/AKT) played a key role in the establishment and maintenance of neurite polarity (Gobrecht et al., 2016; Ranea-Robles et al., 2018). WB results showed that PTL prominently increased the expression of Shh and inhibited PI3K/AKT/GSK3 signaling in a time-dependent manner in C8-D1A cells (Figure 6F). GSK phosphorylation, which depended on PI3K/AKT signaling, inhibited most of these microtubule-associated proteins (MAPs), which regulated axon growth (Chiang et al., 2009).

These results suggested that PTL might affect Shh expression *via* the PI3K/AKT/GSK3 pathway, promoting the mobilization of NEPs after irradiation. Therefore, we used AKT agonist (SC-79), AKT inhibitor (Triciribine, TCN) and PI3K inhibitor (LY294002, LY) to validate the mechanism by which PTL promotes Shh expression in C8-D1A. We found Shh expression was inhibited by SC-79 inhibited and increased by TCN and LY, suggesting that inhibition rather than activation of the PI3K/AKT pathway upregulates Shh in astrocytes. PTL was able to reverse the inhibitory effect of SC-79, and PI3K/AKT activators TCN, LY and PTL synergistically promoted Shh expression (Figure 6G). To verify the regulatory effect of PI3K/AKT pathway on Shh expression *in vivo*, radiation protection experiments in mice were carried out with TCN, SC79 and PTL. Interestingly, perhaps because of the complex conditions *in vivo*, IF assays showed that cerebellar Shh was upregulated after treatment with TCN alone without irradiation whereas PTL treatment alone had no significant effect on it. The TCN-treated mice in our experiments did not survive into adulthood. Consistent with the results of *in vitro* experiments, PTL or TCN increased the expression of Shh in the cerebellum after irradiation. Even *in vivo*, although the effect of PTL treatment was weaker than that of control group, PTL treatment could reverse the inhibition of SC79 (Figure 6H). These results suggest that PTL can not only protect astrocytes from radiation damage, but also promote the secretion of Shh by irradiated astrocytes by inhibiting the PI3K/AKT pathway.

## Discussion

Generally, mammalian neurons do not regenerate after injury, which results in functional deficits. Although significant progress has been made in understanding the mechanisms of neuronal regenerative failure, there is no specific neuroregenerative therapy at present (Varadarajan et al., 2022). The ideal treatment for nerve injury should promote neuron regeneration while inhibiting cellular susceptibility to injury and cellular damage. PTL is a sesquiterpene lactone derived from the herb feverfew, which is traditionally used as medicine. As a covalently active compound, it exhibits anti-inflammatory, redox-regulatory, epigenetic and selective cytotoxicity against tumor stem and progenitor cells (Goodrich et al., 1997). Various published studies have focused on the antitumor activity of PTL. However, more recently, PTL has been shown to exhibit other bioactivities besides its classical function. At very low concentrations, PTL significantly accelerated nerve growth and regeneration by inhibiting microtubule detyrosine in damaged neurons (Diekmann and Fischer, 2016; Freund et al., 2019). In the present study, we utilized irradiation to induce injury to investigate the effect of PTL on cerebellum regeneration. It has been long known that reactive oxygen species (ROS) produced in the cells following irradiation are key mediators of oxidative damage to the cells. The Nrf2-Keap1 pathway is highly sensitive to ROS-dependent oxidative stress in cells. When Keap1 detects ROS, it releases Nrf2, which is then translocated from the cytoplasm to the nucleus. When activated, Nrf2 counteracts ROS production and cell survival (Bader et al., 2021). Previous studies suggest that PTL can increase the radiosensitivity of tumor cells and protect normal cells through its different effects on the KEAP1-NRF2 pathway (Xu et al., 2013). In this study, we first showed the protective effect of PTL on developing cerebellum with acute irradiation injury. γ-H2AX and cleaved caspase3 are proteins formed during irradiation-induced DNA damage and apoptosis, respectively. γ-H2AX and cleaved caspase3 staining showed reduced apoptosis and decreased damage to GNPs. Intracellular H_2_O_2_ levels can reasonably reflect general changes in intracellular ROS production during the conversion of various intracellular ROS to H_2_O_2_; therefore, selective measurement of H_2_O_2_ is essential for accurate assessment of oxidative stress (Calabria et al., 2020). Our results indicated that PTL can alleviate irradiation-induced oxidative stress by reducing H_2_O_2_ levels. Through *in vitro* cell experiment, we confirmed that PTL can provide radiation protection by reducing ROS, inhibiting KEAP1, promoting nuclear translocation of Nrf2 and reducing apoptosis. Previous reports on the relationship between PTL and ROS are still contradictory and incongruent. Some studies have indicated that PTL induces apoptosis *via* ROS generation and blockade of components of the intrinsically anti-apoptotic NF-κB pathway, including its downstream regulator IκB kinase. Other animal experiments have suggested that PTL displays anti-inflammatory effects by inhibiting ROS production *in vivo* (Freund et al., 2020; Wang et al., 2020). Data mostly from previous *in vitro* experiments using cultured cell lines with higher concentrations demonstrated that PTL increased ROS-induced apoptosis. In contrast, the results from normal cells as well as *in vivo* experiments mostly demonstrated its cytoprotective effect. We speculate that this apparent difference may be due to a more complex cellular environment *in vivo*, including the presence of regulatory networks in different types of cells. As early as 1955, it was reported that in irradiated cerebellum, glucose oxidative conversion was somewhat disturbed and the effective glycolytic process was relatively poor (Golubtsova, 1956). It is known that, during aerobic oxidation, reactive oxygen species (ROS) are produced. Aerobic glycolysis can reduce the ROS produced by the respiratory chain of mitochondria during oxidative phosphorylation (OXPHOS), and reduce the oxidative stress burden of cells. (Pascale et al., 2020). PKM2 is a key regulator of aerobic glycolysis, and as an activator of PKM2, PTL may reduce ROS production to some extent by maintaining aerobic glycolysis in the proliferating GNPs. Our data are consistent with the results of previous studies on the reduction of normal cell irradiation sensitivity by PTL *via* the ROS/KEAP1/NRF2 pathway. Besides, some studies revealed that blocking the nuclear translocation of PKM2 and inhibiting the expression of apoptosis-regulated genes can alleviate AD neuronal lesions (Traxler et al., 2022), and PTL may also play an anti-apoptotic role by promoting the formation of PKM2 tetramers and inhibiting dimerization into the nucleus. Above all, PTL is potentially protective against irradiation-induced acute cerebellum injury.

In addition, the exploration of therapeutic strategies targeting neural regeneration may be more important, as increased regeneration is a more likely to attenuate permanent nerve deficits. Our previous study identified a novel population of progenitors in the developing cerebellum EGL that overexpressing Nestin. These NEPs are committed to the granule neuron lineage. In comparison with GNPs, NEPs express fewer genes associated with DNA repair, and exhibit increased genomic instability and tumorigenic potential (Li et al., 2013). In 2017, Wojcinski and colleagues revealed that Nestin-expressing cerebellar progenitor cells in the Purkinje cell layer form only interneurons and astroglia during normal development, expand their differentiation capacity, and originate as mature granule neurons after injury (Wojcinski et al., 2017). We suggest that the differences in location and time of the observations likely account for the seemingly conflicting results on NEPs lineage restriction. While destination of NEPs in inner-EGL was the main concern in our previous work, Wojcinski mainly focused on NEPs in the PCL. Indeed, the latter study also recognized that NEPs in PCL migrate to EGL to become committed to a neuronal lineage after being activated by injury. Together, these studies indicate that NEPs is a unique population of progenitors essential for regenerating GNPs after cerebellar injury. Therapeutic strategies that target activation of NEPs contribute to regeneration and functional recovery of the damaged developing cerebellum. By comparing the size and GNPs proliferation status of the cerebellum at different developmental stages, we confirmed that PTL pretreatment promoted the repopulation of the cerebellum after irradiation. Using transgenic Nestin-CFP mice, we found that a single PTL pretreatment alone could increase the NEPs in EGL and PCL of irradiated developing cerebellum. Also, in agreement with the results of Wojcinski. et al., we found that irradiation induced the appearance of SOX2+ cells in the ML and EGL, whereas PTL significantly increased SOX2+ cells in these regions. The SOX2 gene plays a critical role in regulating neural development, as well as in self-renewal of embryonic and neural stem cells (Jagga et al., 2021). Endogenous expression of Sox2 in neural progenitor cells has a role in promoting reprogramming (Wojcinski et al., 2017). These results suggest that PTL can facilitate the initial adaptive reprogramming of NEPs or promote the migration of NEP-derived cells to EGL after irradiation. Pax6 staining and lineage tracing analysis also verified that PTL contributes to the replenishment of damaged cerebellar granule neuronal cells. Although it has been suggested that NEPs in PCL can normally only differentiate into interneurons and glial cells, probably because of experimental methodological differences, we still found that a small amount of PAX6 positive NEPs exists in the normal cerebellum, which is consistent with the lineage tracing experiment. In contrast to single irradiation that reduces the expression of the interneuron marker PAX2 in NEPs, PTL pretreatment protects against the inhibitory effect of irradiation on interneurons derived from NEPs. This helps maintain the balance of excitatory and inhibitory neuronal cells in the microenvironment. PTL also exerts the same effect on glial cells to reverse the effects of irradiation and maintain homeostasis. These results suggest that PTL may have the ability not only to promote the reprogramming and differentiation of NEPs into granule neuron cells after irradiation, but also to expand NEPs cells and maintain their role of producing interneurons and glial cells. That is, although the cell lineage of NEPs is somewhat controversial, PTL mobilizes both NEPs located in the EGL and PCL, and may act on the most essential and basic level. Energy metabolism is a fundamental physiological activity in cells and individuals. Besides affecting the adaptive reprogramming capacity of brain progenitor cells, PTL may also regulate the energetic reprogramming of progenitor cells through its effects on PKM2. Katherine et al. (Tech et al., 2017) confirmed that PKM2 was highly expressed in developing cerebellar progenitor cells. GNPs with PKM2 deletion showed a decrease in lactic acid production and an increase in SHH-driven proliferation. The allosteric regulation of PKM2 activity is the basis of its effect on metabolic reprogramming. As a tetramer activator of PKM2, PTL may inhibit the glycolysis of PKM2 and regulate NEPs mobilization.

Previous studies have suggested that both NEPs in the PCL and a small number of scattered NEPs in the EGL are affected by Shh signaling after activation. Therefore, we believe that investigating Shh signaling could help elucidate PTL-promoted amplification of cerebellar NEPs after irradiation. Our *in vitro* experiments revealed that PTL itself neither promotes nor inhibits the response of GNPs to Shh, implying that it has no direct effect on Shh signaling. Shh was detected in cerebellar tissue at different time points, suggesting that although PTL is not an activator or inhibitor of Shh, it can promote Shh production in the cellebellar microenvironment. DISP1 is indispensable in cholesterol bio-synthesis or secretion of cholesterol-modified Shh (Jamal et al., 2018). Yuan et al. (Yuan et al., 2020) found that PTL can affect the TCA cycle, amino acid metabolism, choline metabolism and lipid metabolism in thyroid cancer cells (TPC-1). Our data show that PTL increases DISP1 expression, contributing to the secretion of Shh. As the primary source of cerebellar Shh, Purkinje cell neurogenesis is synchronized with cerebellar development and begins at E10.5. Compared with prenatal irradiation, postnatal irradiation had slightly less effect on cerebellar Purkinje cells (Zhou et al., 2017). We found that PTL could upregulate Shh in irradiation-induced cerebellar Purkinje cells to some extent. Astrocyte is the major site of acute aerobic glycolysis in brain (Barros et al., 2021). Certain conditions induce astrocytes to produce Shh. It has been found that reactive astrocytes secrete Shh to promote the proliferation of astrocytes, microglia and NG2 positive progenitor cells (Pitter et al., 2014). Various CNS injury factors, including irradiation, can lead to an increase in reactive astrocytes (Liddelow and Barres, 2017). We found that PTL increases astrocyte number and Shh expression. Given that astrocytes are more sensitive to irradiation and PTL than Purkinje cells, we explored the possible mechanisms of PTL-induced astrocyte-associated Shh production. Regarding the relationship between astrocytes and Shh, studies have mostly focused on determining the important role of the Shh signaling pathway (Allahyari et al., 2019) and less on the regulation of Shh protein expression itself. The controllable temporospatial expression of Shh is the basis of its normal function. Multiple signaling pathways are associated with the regulation of Shh expression, and positive and negative transcriptional regulatory networks control its expression in restricted regions (Matsubara et al., 2017). Numerous tumor types are associated with dysregulation of the Shh signaling pathway, including constitutive overexpression of the Shh ligand (Tolani et al., 2018). Although the effects of PTL are mostly associated with its role as an NF-κB inhibitor, some studies have proposed that the overexpression of Shh in breast cancer is due to NF-κB activation (Cui et al., 2010), which apparently cannot explain the phenomenon that PTL promotes Shh expression. As previously described, PTL protects the peripheral nerve in an NF-κB-independent manner, and it mimics the beneficial effects of GSK3 mutations on axon regeneration in mice. In the exploration of the inhibition of Wnt/β-catenin signal by PTL, researchers found that PTL can reverse the inhibitory effect of GSK3 ion downstream genes (Zhu et al., 2018). GSK-3 is a key regulator of neuronal progenitor homeostasis. GFAP-cre-mediated deficiency of GSK-3 in astrocytes leads to enlarged brains and more and larger astrocytes (Jung et al., 2016). The PI3K/Akt is a major signaling pathway that regulates GSK3. Numerous studies have shown that the PI3K-Akt-GSK3β signal transduction pathway plays important roles in the neuroprotective process. Our results confirmed that PTL suppressed activation of PI3K/Akt signaling in astrocytes. The PI3K/Akt pathway has crosstalk with multiple cells signaling pathways. Activation of STAT3 is thought to upregulate Shh gene expression (Zagozewski et al., 2022), whereas PI3K/AKT inhibition promotes STAT3 activation. Recently, RARβ2, another promoter of Shh expression, was also found to be negatively regulated by PI3K/AKT (Huang et al., 2022). Through *in vitro* and *in vivo* experiments of PI3K/AKT inhibitor, we also confirmed that it can promote the expression of Shh in cells and irradiated cerebellar tissue. Surprisingly, a single administration of PTL *in vivo* did not promote cerebellar Shh expression. Our previous studies found that the homeostasis of NEPs is important. Under normal conditions, NEPs are quiescent *in vivo*, physiological concentrations of Shh do not trigger their mobilization, and abnormal proliferation of NEPs is associated with tumorigenesis. PTL functions only in the presence of abnormalities or injury, and is greatly likely to be attributable to changes in aerobic glycolysis, which facilitates the maintenance of homeostasis. Previous studies also confirmed that the Shh signaling pathway and PI3K/AKT synergize with each other to affect aerobic glycolysis of GNPs in the developing cerebellar EGL (Gershon et al., 2013; Tech et al., 2017). On the other hand, this suggests that the role of PTL on PI3K/AKT may be complex, especially since many studies have shown that PTL exert effects on various signaling pathways. Akt is a key kinase that promotes growth, metabolism, survival, and motility. Although further studies are needed to determine the specific molecular mechanisms by which PTL, PI3K/AKT and Shh regulate each other, these results are still encouraging.

## Conclusion

Our work shows that PTL exerts a protective effect on irradiation-induced acute injury through the ROS/KEAP1/NRF2 pathway, and facilitates the expansion and reprogramming of NEPs in the damaged cerebellum, enhancing the regeneration of the damaged cerebellum by promoting Shh expression in irradiated cerebellar tissue. These findings suggest that therapeutic strategies based on PTL may provide new options for treatment after CNS injury, especially since PTL not only provides a protective effect on the damaged nerve cells themselves, but also facilitates nerve cell regeneration by affecting the microenvironment. Considering that PTL can also promote axonal regeneration of peripheral nerve cells and affect aerobic glycolysis, elucidating the metabolic mechanism of the effect of PTL on astrocyte Shh through PI3K/Akt inhibition, the effects of PTL on other cells (such as Purkinje cells, oligodendrocytes and microglia) and axonal regeneration of CNS neurons and the regulation of energy metabolism in NEPs warrant further study.

## Summary

Given the multiple effects of PTL on the developing cerebellum after injury, which modulates both the injured cells and the microenvironment in which they reside, our findings provide a rationale for PTL as a potential CNS radioprotective agent.

## Data Availability

The original contributions presented in the study are included in the article/Supplementary Material, further inquiries can be directed to the corresponding author.
