# Supplementary material for: Parthenolide promotes expansion of Nestin+ progenitor cells via Shh modulation and contributes to post-injury cerebellar replenishment
Source: Front Pharmacol. 2022 Oct 28;13:1051103. doi: 10.3389/fphar.2022.1051103 (PMC9651157; doi:10.3389/fphar.2022.1051103)
Supplement: Supplementary file 1 [file DataSheet1.docx]

**Supplementary**

**S1. Effect of TAM administration at different time points on NEPs labeled with GFP in NestinCreERT2 × ROSA26-LSL YFP mice**

**
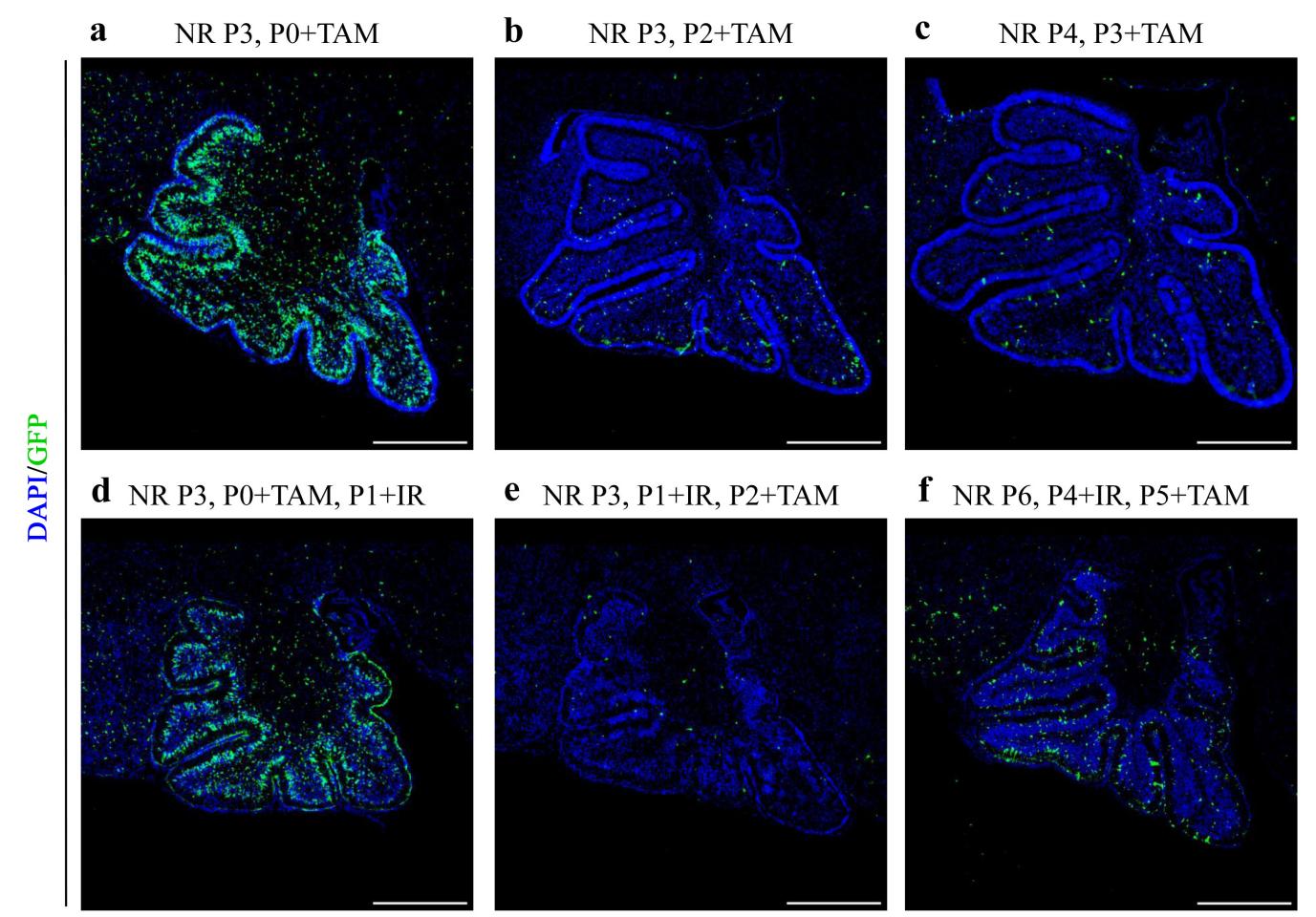
**

a. GFP and DAPI staining in the P3 midsagittal section of the cerebellum of NR mice administered TM at P0 without IR.

b. GFP and DAPI staining in the P3 midsagittal section of the cerebellum of NR mice administered TM at P2 without IR.

c. GFP and DAPI staining in the P4 midsagittal section of the cerebellum of NR mice administered TM at P3 without IR.

d. GFP and DAPI staining in the P3 midsagittal section of the cerebellum of NR mice administered TM at P0 and received IR at P1.

e. GFP and DAPI staining in the P3 midsagittal section of the cerebellum of NR mice received IR at P1 and administered TM at P2.

f. GFP and DAPI staining in the P6 midsagittal section of the cerebellum of NR mice received IR at P4 and administered TM at P5.

**S2. Effects of intraperitoneal injection of PTL at different doses and exposure times on EGL cell proliferation and DNA damage**

**
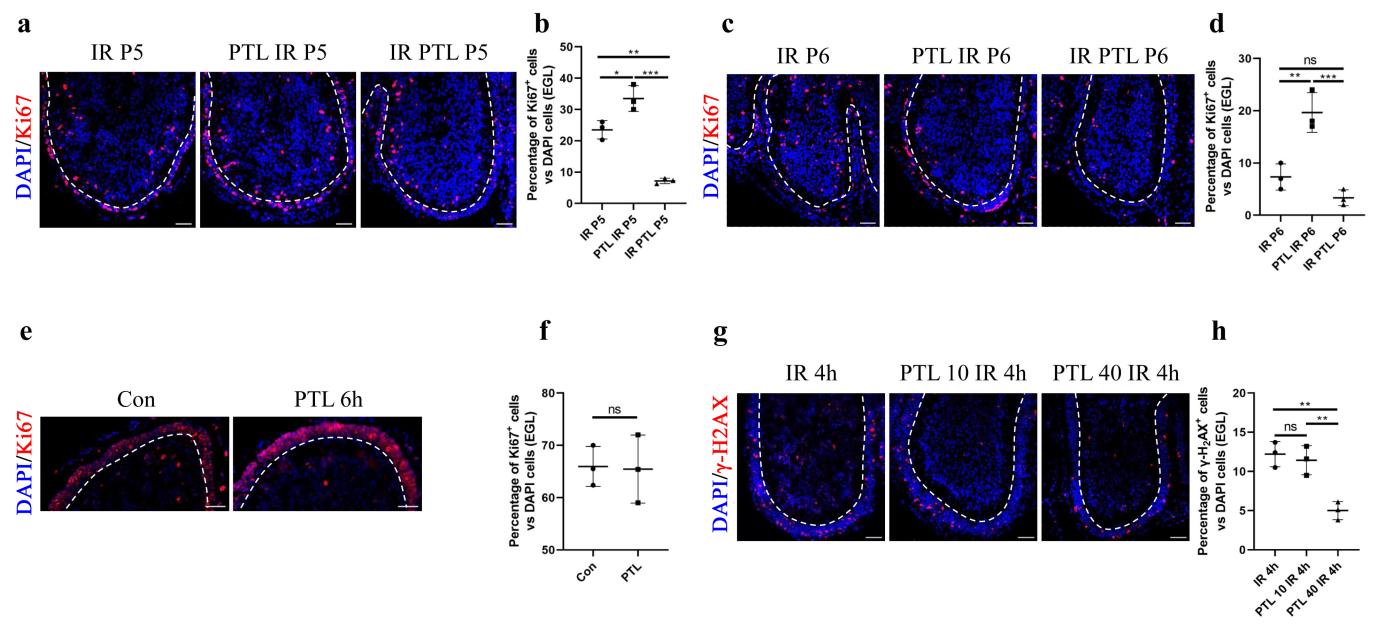
**

1. IF detection of Ki67 and DAPI on sagittal sections of Con and PTL mice.
2. Graph indicating the percentage of Ki67+ cells relative to all DAPI-positive cells of Con and PTL mice in EGL by 6 hours.
3. IF detection of Ki67 and DAPI on sagittal sections of IR, PTL IR and IR PTL mice on 24 hours after IR at P4.
4. Graph of number of Ki67+ cells vs all DAPI cells in EGL of IR, PTL IR and IR PTL mice by 24 hours after IR at P4.
5. IF detection of Ki67 and DAPI on midsagittal sections of IR, PTL IR and IR PTL mice on 48 hours after IR at P4.
6. Graph indicating the percentage of Ki67+ cells relative to all DAPI-positive cells in EGL of IR, PTL IR and IR PTL mice 48 hours after IR at P4.
7. IF detection of γ-H2AX and DAPI on midsagittal sections of IR, pre-treated with 40mg/kg PTL IR and pre-treated with 10mg/kg PTL IR mice by 4 hours after IR at P4.
8. Graph of number of Ki67+ cells vs all DAPI cells in EGL of IR, pre-treated with 40mg/kg PTL IR and pre-treated with 10mg/kg PTL IR mice by 4 hours after IR at P4.

The external granule cell layer (EGL) painted by white dotted line. All graphical data were presented as mean ± s.e.m., and significance was determined by two-tailed Student’s t test or Kruskal–Wallis nonparametric one-way ANOVAs. ns, nonsignificant; *P < 0.05; **P < 0.01; ***P < 0.001. White scale bar, 50 µm.

**S3. PTL increase irradiated cerebellum PAX6+ cells in different transgene mice**

**
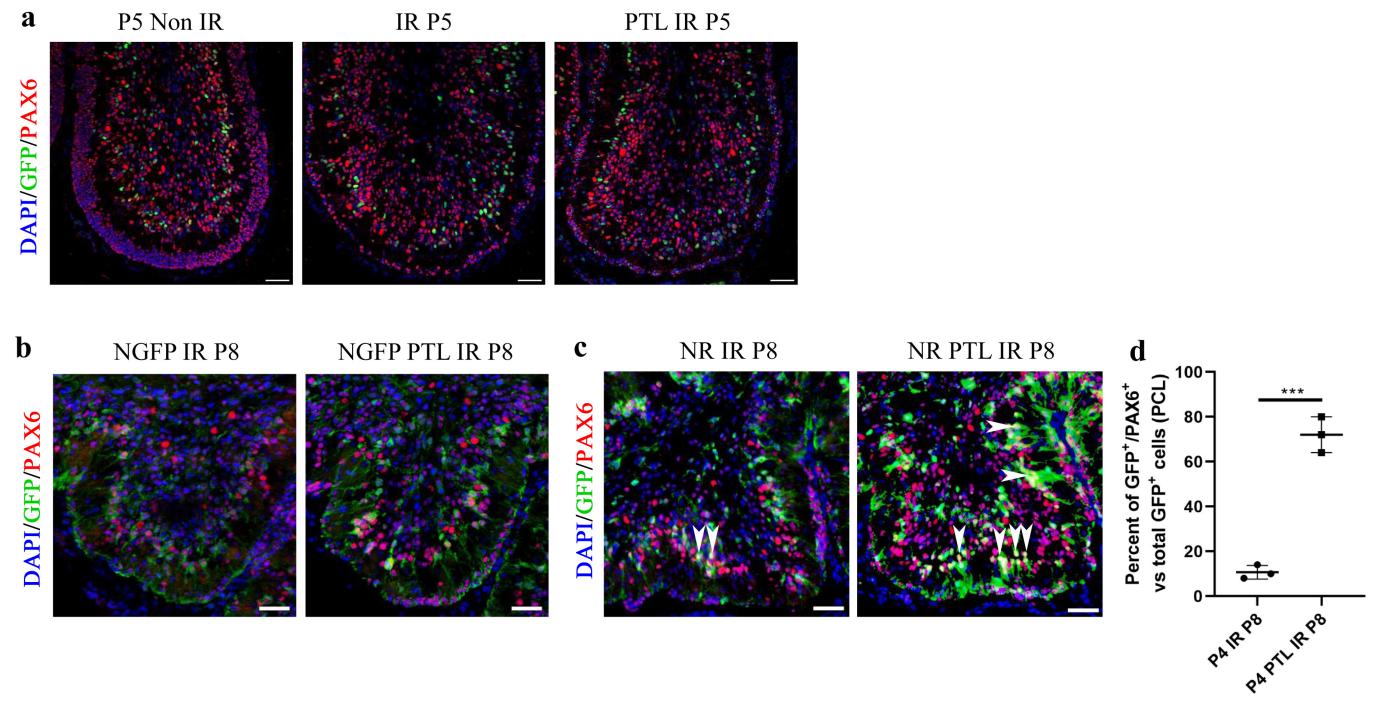
**

a. IF detection of GFP, PAX6 and DAPI on midsagittal sections of Non IR, IR and PTL IR mice at P5.

b. IF detection of GFP, PAX6 and DAPI on midsagittal sections of IR and PTL IR mice at P8 in Nestin-GFP mice.

c. IF detection of GFP, PAX6 and DAPI on midsagittal sections of IR and PTL IR mice at P8 in NR mice. White arrowheads indicated GFP+/PAX6+ cells in PCL.

d. Graph of number of GFP+/PAX6+ cells vs all GFP cells in PCL of IR and PTL IR mice at P8 in NR mice.

All graphical data were presented as mean ± s.e.m., and significance was determined by two-tailed Student’s t test or Kruskal–Wallis nonparametric one-way ANOVAs. ns, nonsignificant; *P < 0.05; **P < 0.01; ***P < 0.001.

**S4. Effect of PTL on cerebellar Purkinje cells after irradiation**

**
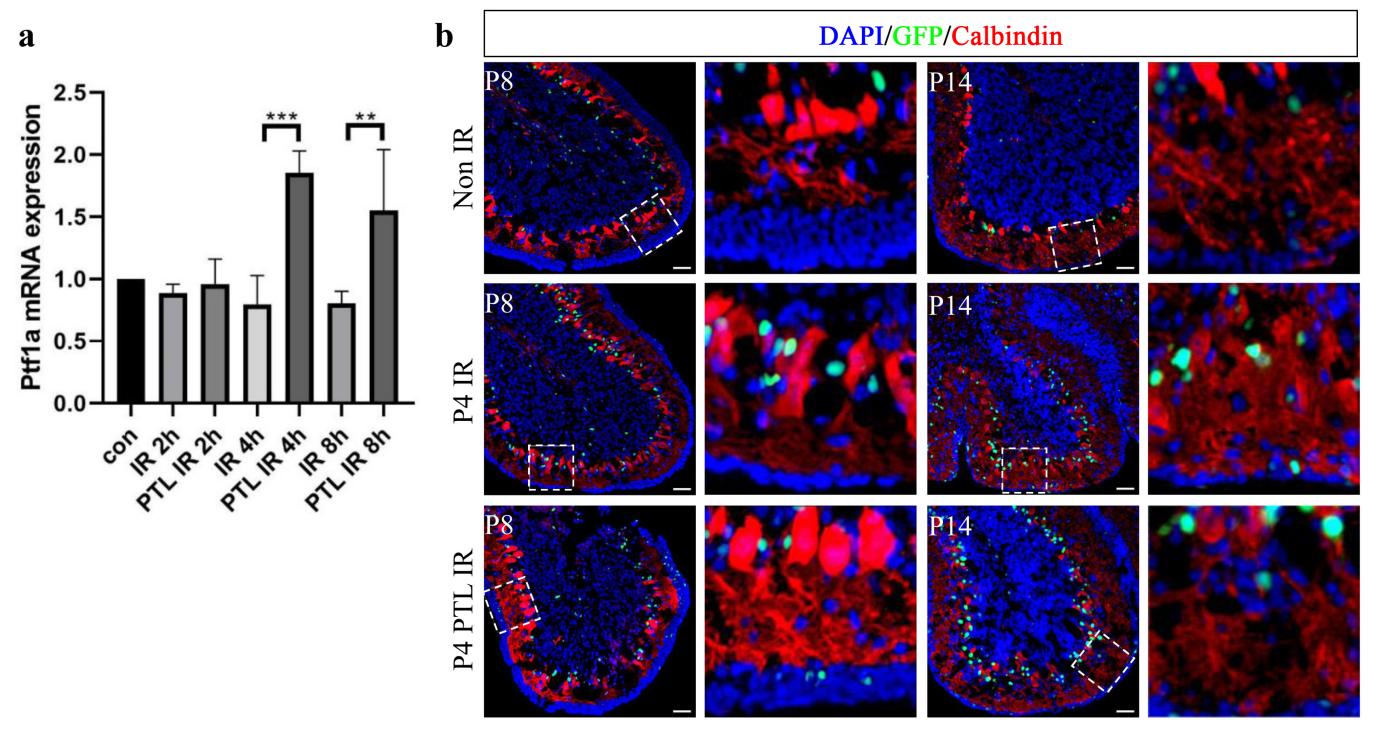
**

a. QRT-PCR of Ptf1a from the cerebellum of wild type mice pre-treated with 40mg/kg PTL or not at 2, 4, 8 hours after irradiation with 4Gy X-ray.

b. IF detection of GFP, Calbindin and DAPI on midsagittal sections of Non IR, IR and PTL IR mice at P8 and P14. High-power images were shown of the areas indicated by white dotted rectangles.

**Table S1 Animal groups researched in this study.**

| **Mice** | **Groups** | | | | |
| --- | --- | --- | --- | --- | --- |
| C57BL/6J | P4 IR 4h | P4 PTL IR 4h |  |  |  |
| C57BL/6J | P4 Con | P4 PTL | P4 IR 2h | P4 PTL IR 2h |  |
| C57BL/6J | P5 Con | P4 IR 24h | P4 PTL IR 24h |  |  |
| C57BL/6J | P8 Con | P4 IR P8 | P4 PTL IR P8 |  |  |
| C57BL/6J | P14 Con | P4 IR P14 | P4 PTL IR P14 |  |  |
| C57BL/6J | P30 Con | P4 IR P30 | P4 PTL IR P30 |  |  |
| Nestin CFP | P5 Con | P4 IR 24h | P4 PTL IR 24h |  |  |
| Nestin CFP | P8 Con | P4 IR P8 | P4 PTL IR P8 |  |  |
| Nestin CFP | P14 Con | P4 IR P14 | P4 PTL IR P14 |  |  |
| Nestin creER^T2^, Rosa26 LSL YFP | P4 IR P21 | P4 PTL IR P21 |  |  |  |
| Nestin creER^T2^, Rosa26 LSL YFP | P0+TAM, P3 | P2+TAM, P3 | P3+TAM, P4 |  |  |
| Nestin creER^T2^, Rosa26 LSL YFP | P0+TAM, P1 IR, P3 | P1 IR, P2+TAM, P3 | P4 IR, P5+TAM P6 |  |  |
| C57BL/6J | P4 Con | P4 PTL 6H |  |  |  |
| C57BL/6J | P4 IR 24h | P4 PTL IR 24h | P4 IR PTL 24h |  |  |
| C57BL/6J | P4 IR 48h | P4 PTL IR 48h | P4 IR PTL 48h |  |  |
| Nestin GFP | P4 IR P8 | P4 PTL IR P8 |  |  |  |
| C57BL/6J | P4 CON | P4 IR 2H | P4 IR 4H | P4 IR 8H |  |
| C57BL/6J |  | P4 PTL IR 2H | P4 PTL IR 4H | P4 PTL IR 8H |  |

**Table S2 Primary antibodies used for IF in this study.**

| **Name** | **Manufactor** | **Cat.No** | **Ratio** |
| --- | --- | --- | --- |
| GFP | Abcam | ab13970 | 1:500 |
| Ki67 | Abcam | ab15580 | 1:500 |
| Shh | Abcam | Ab50515 | 1:300 |
| GFAP | Cell Signaling Technology | 12389 | 1:300 |
| Tuj1 | CST | 5568 | 1:500 |
| PAX6 | Proteintech | 12323-1-AP | 1:300 |
| NeuN | Proteintech | 26975-1-AP | 1:300 |
| SOX2 | Proteintech | 11064-1-AP | 1:300 |
| Calbindin | Proteintech | 66394-1-Ig | 1:300 |
| S100β | Zen BioScience | 380829 | 1:300 |
| PAX2 | Zen BioScience | 380838 | 1:300 |
| γ-H2AX | Zen BioScience | 320015 | 1:200 |
| Cleaved-caspase3 | Beyotime | AC033 | 1:200 |

**Table S3 Secondary antibodies used for IF in this study.**

| **Name** | **Manufactor** | **Ratio** |
| --- | --- | --- |
| Alexa Fluor-594 anti-rabbit IgG | Jackson Immuno Research | 1:200 |
| Alexa Fluor 488 anti-chicken IgY | Jackson Immuno Research | 1:500 |
| Cy3 anti-rabbit IgG | Proteintech | 1:300 |
| Cy3 anti-mouse IgG | Proteintech | 1:300 |
| Alexa Fluor 488 anti-rabbit IgG | Proteintech | 1:300 |
| Alexa Fluor 488 anti-mouse IgG | Proteintech | 1:300 |

**Table S4 Primer pairs used in this study.**

| **Name** | **Sequences** |
| --- | --- |
| GAPDH- Forward | TGGATTTGGACGCATTGGTC |
| GAPDH- Reverse | TTTGCACTGGTACGTGTTGAT |
| Shh- Forward | AAAGCTGACCCCTTTAGCCTA |
| Shh- Reverse | TTCGGAGTTTCTTGTGATCTTCC |
| Ptf1a-Forward | TCCCATCCCCTTACTTTGATGA |
| Ptf1a-Reverse | GTAGCAGTATTCGTGTAGCTGG |
| DISP1-Forward | ACCCTGGGGAATTACATCGC |
| DISP1-Reverse | GTGCCGTTTTGGTAGTGCTT |

**Table S5 Primary antibodies used for WB in this study.**

| **Name** | **Manufactor** | **Cat.No** | **Ratio** |
| --- | --- | --- | --- |
| Caspase3 | Proteintech | 19677-1-AP | 1:1000 |
| Cleaved-Caspase3 | Beyotime | AC033 | 1:1000 |
| BCL2 | Proteintech | 12789-1-AP | 1:1000 |
| BAX | Proteintech | 60267-1-Ig | 1:1000 |
| PARP | Proteintech | 13371-1-AP | 1:1000 |
| Shh | Sigma | S4944 | 1:2000 |
| Keap1 | Proteintech | 60027-1-Ig | 1:1000 |
| Nrf2 | Zen BioScience | 340675 | 1:1000 |
| AKT | Proteintech | 60203-2-Ig | 1:1000 |
| p-AKT | Zen BioScience | 381555 | 1:1000 |
| PI3K | Proteintech | 60225-1-Ig | 1:1000 |
| p-PI3K | Beyotime | AF5905 | 1:1000 |
| GSK3β | Beyotime | AF1543 | 1:1000 |
| p-GSK3β | Beyotime | AF5830 | 1:1000 |
| α-Tubulin | Proteintech | 66031-1-Ig | 1:5000 |
| GAPDH | Proteintech | 60004-1-Ig | 1:5000 |
| Laminin B1 | Proteintech | 66095-1-Ig | 1:5000 |
|  |  |  |  |

**Table S6 Secondary antibodies used for WB in this study.**

| **Name** | **Manufactor** | **Ratio** |
| --- | --- | --- |
| Peroxidase-conjugated Affinipure Goat anti-Rabbit IgG | Proteintech | 1:5000 |
| Peroxidase-conjugated Affinipure Goat anti-Mouse IgG | Proteintech | 1:5000 |
| HRP Goat Anti-Chicken IgY | Beyotime | 1:5000 |
| HRP Anti-Rat IgG | Beyotime | 1:5000 |
